# Phenomic and transcriptomic analyses reveal the sequential synthesis of Fe_3_O_4_ nanoparticles in *Acidithiobacillus ferrooxidans* BYM

**DOI:** 10.1128/spectrum.01729-23

**Published:** 2023-10-06

**Authors:** Jiani Yang, Shuang Zhang, Yu Zhang, Dan Zhao, Tao Liu, Xindi Sun, Lei Yan

**Affiliations:** 1 Heilongjiang Provincial Key Laboratory of Environmental Microbiology and Recycling of Argo-Waste in Cold Region, Heilongjiang Bayi Agricultural University, Daqing, Heilongjiang, China; 2 College of Life Science and Biotechnology, Heilongjiang Bayi Agricultural University, Daqing, Heilongjiang, China; 3 Key Laboratory of Low-Carbon Green Agriculture in Northeastern China, Ministry of Agriculture and Rural Affairs, Daqing, Heilongjiang, China; University of Minnesota Twin Cities, St. Paul, Minnesota, USA

**Keywords:** *Acidithiobacillus ferrooxidans*, iron biomineralization, magnetite magnetosomes, transcriptomics, molecular mechanism

## Abstract

Understanding the molecular mechanism of magnetite (Fe_3_O_4_) nanoparticle synthesis in *Acidithiobacillus ferrooxidans* BYM is particularly important for the commercial development of biogenic Fe_3_O_4_ nanoparticles. The phenomic parameters such as intracellular iron content and number and size of Fe_3_O_4_ nanoparticles were significantly affected by different treatment conditions, i.e., FeSO_4_·7H_2_O concentrations (0, 40, and 80 g/L), growth times (12, 36, and 50 h), and magnetic field intensities (0.05, 3.5, and 15 mT) (*P* < 0.01). Transcriptome analysis revealed that 2,164, 1,587, and 1,061 differentially expressed genes (DEGs) were accordingly detected, and 24 significant expression profiles were identified in *A. ferrooxidans* BYM under the three treatment conditions. The construction of gene regulatory networks for Fe_3_O_4_ nanoparticle synthesis indicated that DEGs mainly enrich ion transport, oxidation-reduction process, membrane structure, signal transduction, and quorum sensing. The four modules were found to be significantly associated with Fe_3_O_4_ nanoparticle phenomic parameters using a weighted gene co-expression network. Ten hub genes significantly correlated with Fe_3_O_4_ nanoparticle phenomic parameters (*P* < 0.01) were finally selected from 24 eigengenes related to iron metabolism screened from these models. On the basis of the previous research results and the present study findings, we provide a hypothetical molecular model for Fe_3_O_4_ nanoparticle synthesis mediated by these hub genes in *A. ferrooxidans* BYM comprising membrane formation, iron uptake and transport, iron redox, and crystal maturity. Our results will enable in-depth studies of Fe_3_O_4_ nanoparticle synthesis in non-magnetotactic magnetosome-producing bacteria.

**IMPORTANCE:**

As the most important non-magnetotactic magnetosome-producing bacteria, *Acidithiobacillus ferrooxidans* only requires very mild conditions to produce Fe_3_O_4_ nanoparticles, thus conferring greater flexibility and potential application in biomagnetic nanoparticle production. However, the available information cannot explain the mechanism of Fe_3_O_4_ nanoparticle formation in *A. ferrooxidans*. In this study, we applied phenomic and transcriptomic analyses to reveal this mechanism. We found that different treatment condition factors notably affect the phenomic data of Fe_3_O_4_ nanoparticle in *A. ferrooxidans*. Using transcriptomic analyses, the gene network controlling/regulating Fe_3_O_4_ nanoparticle biogenesis in *A. ferrooxidans* was proposed, excavating the candidate hub genes for Fe_3_O_4_ nanoparticle formation in *A. ferrooxidans*. Based on this information, a sequential model for Fe_3_O_4_ nanoparticle synthesis in *A. ferrooxidans* was hypothesized. It lays the groundwork for further clarifying the feature of Fe_3_O_4_ nanoparticle synthesis.

## INTRODUCTION

Magnetotactic bacteria (MTB), which are widespread across distantly related bacterial phyla, belong to the 13 phyla based on the Genome Taxonomy Database Toolkit (1). They are a class of microorganisms that produce magnetosomes composed of Fe_3_O_4_ or greigite (Fe_3_S_4_) magnetic nanoparticles surrounded by lipid bilayer membranes (2, 3). The magnetosomes gather in a certain location in cells, and most of them usually arrange in chains approximately parallel to the long axis of MTB, thus forming a biomagnetic “compass” (4). MTB could swim to the favorable niche by the geomagnetic field-aided navigation of magnetosome chains and by propulsion of the flagella (5). Some merits endow the biomagnetic nanoparticles with promising potentials in biological and nanotechnological fields, including high stability, good biocompatibility, well dispersibility, and superparamagnetism (6).

As the most important magnetosome-producing bacteria, MTB have attracted more interest since they were discovered, and the studies of MTB have made significant progress in three decades (1, 7). For typical MTB, such as *Magnetospirillum magneticum* AMB-1, *Magnetospirillum magnetotacticum* MS-1, and *Magnetospirillum gryphiswaldense* MSR-1, the concentration of dissolved O_2_ in the growth medium needs to be strictly controlled during incubation (3). The formation of magnetosomes in MTB is strongly inhibited at >5% oxygen. In addition, the presence of polypeptone, yeast extract, and L-cysteine was required for both bacterial growth and magnetosome production (7). The non-magnetotactic magnetosome-producing bacteria widely existed in nature and only received little research attention (8), such as *Acidithiobacillus ferrooxidans*, *Leptospirillum ferrooxidans*, and *Ferroplasma thermophilum*, which can produce a small number of magnetosomes. Among them, *A. ferrooxidans* was found to form similar Fe_3_O_4_ nanoparticles under the conditions of sufficient Fe(II) and a suitable oxygen environment (9, 10). It is an acidophilic autotrophic bacterium with aerobic growth on a low-cost and simple-to-prepare medium (10). The mass cultivation of *A. ferrooxidans* has been easily available with enough Fe(II) (9). Thus, *A. ferrooxidans* is conferring greater flexibility and potential application in biomagnetic nanoparticle production (11).

The magnetic nanoparticle synthesis in magnetosome-producing bacteria is stimulated by various chemical and physical environmental factors (12). One of the important factors is iron substrate (13). Fe(II) or Fe(III) at concentrations between 20 and 50 µM is generally sufficient for cell growth and magnetic nanoparticle formation. It has been reported that the cell growth of *Magnetospirillum* sp. was inhibited once the iron concentrations were over 200 µM (14). Similarly, *A. ferrooxidans* could use Fe(II) under acidic conditions to obtain energy for cell growth and form Fe_3_O_4_ nanoparticles (9). Our previous study indicated that the cell growth and Fe_3_O_4_ nanoparticle formation of *A. ferrooxidans* increased with the increasing Fe(II) concentration in the range of 40–120 and 40–160 mM, respectively (13). In addition, the external magnetic field might also be an important factor affecting the synthesis of magnetic nanoparticles. Under the geomagnetic field, the magnetosomes could generate sufficient torque to push bacteria to swim along geomagnetic field lines (15). It has been found that a near-zero magnetic field (<500 nT) seemed to postpone magnetosome formation and a constant-strength magnetic field (0.2 T) could inhibit the growth of MTB but promote the formation of magnetosomes (16). The pulsed and sinusoidal magnetic fields were also found to promote magnetosome formation but resulted in the uneven particle size and irregular arrangement of the nanoparticles (17). *A. ferrooxidans* exhibited weak magnetotaxis under the action of an applied magnetic field, while its calcined cells with the high Curie temperature indicated the ferromagnetism of magnetosomes (18). However, the effects of the magnetic field on the synthesis of Fe_3_O_4_ nanoparticles have not been revealed (9).

Genomes of several strains of MTB have been completely sequenced. With the development of multi-omic techniques, some genes involved in magnetosome synthesis were identified. It has been reported that the gene clusters comprising >30 genes were found within a chromosome region termed magnetosome island (MAI) consisting of five operons, such as *mamAB*, *mamGFDC*, *mms6*, *mamXY*, and *feoAB* (8, 19). The formation of magnetosomes in MTB is a sequential process that is strictly controlled by genes (20, 21). The proposed model for magnetosome synthesis in α-proteobacterial *M. magneticum* AMB-1 and *M. gryphiswaldense* MSR-1 suggests that magnetosome biogenesis is divided into four stages, i.e., uptake of iron ions, membrane invagination, iron mineralization, and magnetosome chain assembly (7, 22). The hypothetical model of magnetosome biomineralization in δ-proteobacterial *Desulfovibrio magneticus* RS-1 indicates that magnetic crystals are synthesized one at a time from a single magnetosome factory associated with the membrane (22). Thus, the growth time exhibits a significant effect on the formation of magnetosomes in magnetosome-producing bacteria.

As one of the hot topics in microbiology, the mechanistic insights into the magnetosome synthesis are based mainly on the MTB and rarely on non-MTB. The *A. ferrooxidans* BYM genome (3.2 Mb) contains a circular chromosome with 58.54% GC content, 3,260 ORF numbers, three CRISPRs, and a plasmid of 56.44% GC content and 54 ORF numbers (23). Although previous study showed that the several genes of *A. ferrooxidans* shared relatively high similarity with magnetosome genes from MTB, they do not belong to MAI, and no region similar to MAI is found in the genome of *A. ferrooxidans* (23). *A. ferrooxidans* Fe_3_O_4_ nanoparticles are irregularly dispersed in the cells, which is different from the chain arrangement of MTB (10). The mechanism of Fe_3_O_4_ nanoparticle synthesis in *A. ferrooxidans* seems to be different from MTB. However, current researches on Fe_3_O_4_ nanoparticle synthesis in *Acidithiobacillia* spp. were only at the cellular level, but the topic on its molecular mechanism remains understudied (11). It is necessary to find potential key genes by multi-omic analysis and verify these genes by establishing genetic operation system, such as gene overexpression and knockout.

In the present study, the Fe_3_O_4_ nanoparticle phenomic parameters, such as intracellular iron content and number and size of Fe_3_O_4_ nanoparticles, and the transcriptome data of *A. ferrooxidans* BYM were monitored and analyzed under different FeSO_4_·7H_2_O concentrations, growth times, and magnetic field intensities. The hub genes possibly correlated with Fe_3_O_4_ nanoparticle synthesis were identified, and quantitative reverse transcription PCR (qRT-PCR) verified their expression levels. A hypothetical mechanism of Fe_3_O_4_ nanoparticle synthesis was proposed. These findings would broaden our understanding of Fe_3_O_4_ nanoparticle formation in non-magnetotactic magnetosome-producing bacteria.

## MATERIALS AND METHODS

### Bacterial strain, culturing conditions and cell sampling

**FIG 1:**
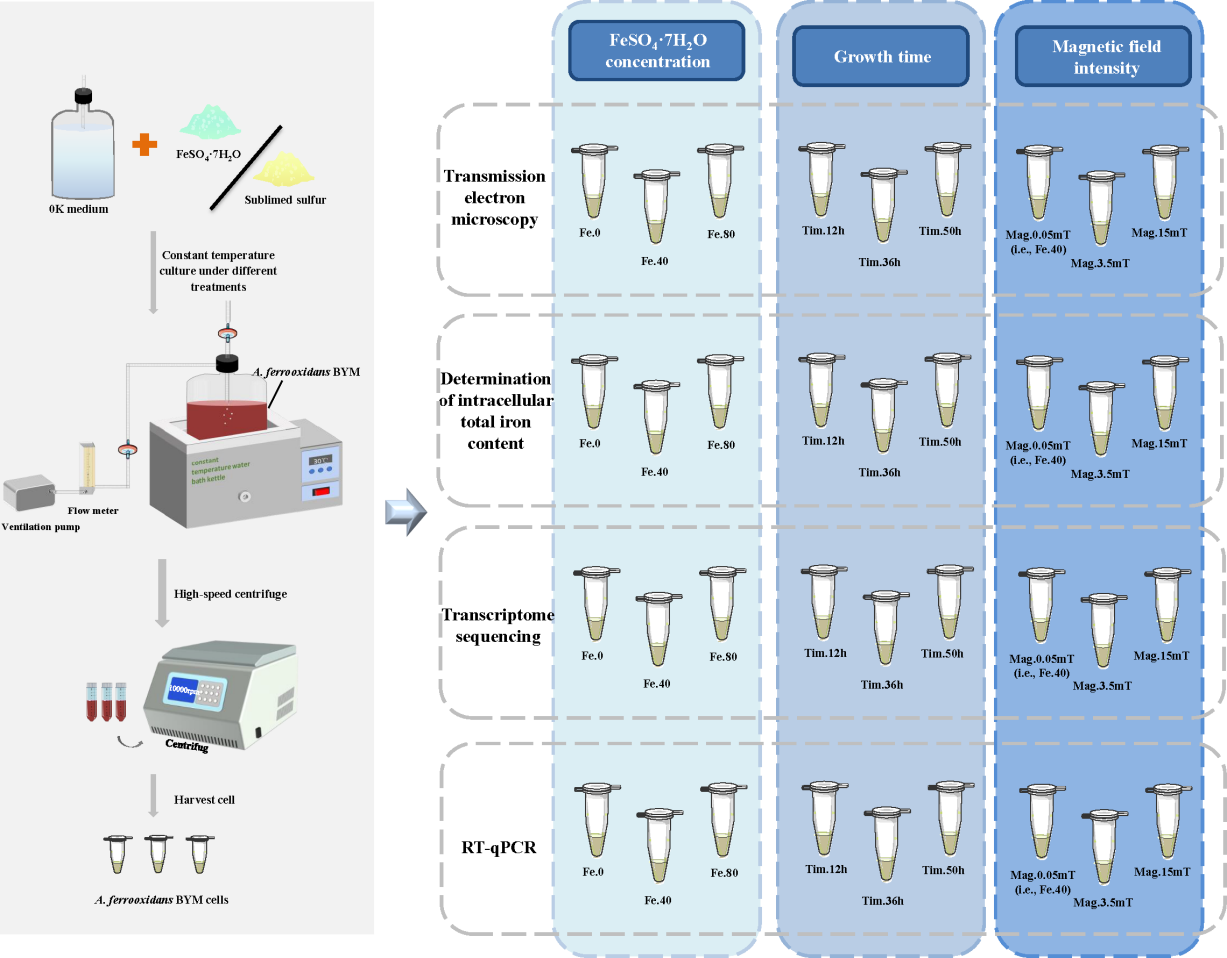
Schematic diagram of sample preparation.

The experimental strain *A. ferrooxidans* BYM, isolated from Baiyin Copper Mine of Gansu Province, was deposited in the China Center for Type Culture Collection (M2018630). *A. ferrooxidans* BYM was cultured in the 0K medium supplied with filter-sterilized FeSO_4_·7H_2_O or sublimed sulfur sterilized by intermittent sterilization under atmospheric pressure. For intermittent sterilization, the sublimed sulfur needs to be heated to 100°C at atmospheric pressure, boiled for 30 min, and repeated three times after cooling. The 0K medium contained 1.2g/L (NH_4_)_2_SO_4_, 0.1 g/L KCl, 0.5 g/L K_2_HPO_4_, 0.5 g/L MgSO_4_·7H_2_O, 2.94 g/L C_6_H_12_O_7_, and 0.01 g/L Ca(NO_3_)_2_ (23). The 0K-FeSO_4_·7H_2_O and 0K-S media were adjusted to pH 1.75 and 4.00 under sterile conditions, respectively. *A. ferrooxidans* BYM was cultivated in a 5-L aerated bioreactor connected with CT-202 air pump (SenSen Group Co., Ltd., Zhoushan, China) and LZB-4WB gas flowmeter (Xiangjin Flowmeter Factory, Xinghua, China) using set ventilation of 0.7 L/min. The temperature (25°C) was controlled using a DK-S24 thermostatic water bath (Senxin Experimental Instrument Co., Ltd., Shanghai, China) (Fig. 1).

*A. ferrooxidans* BYM was inoculated (10%, vol/vol) into 4 L 0K-FeSO_4_·7H_2_O media (24 g/L FeSO_4_·7H_2_O) and 0K-S media (10 g/L sublimed sulfur), respectively. According to the previous report, the logarithmic phase cells were collected as inoculum by filtration and centrifugation (10). In brief, the logarithmic phase cells were filtered by porous metal filter (Chuangwei Filtration Equipment Factory, Haining, China) with the filter membranes (0.22 µm × 50 mM, Xinya Purification Device Factory, Shanghai, China) and then centrifuged for 5 min at 366 and 9,166 × *g*, respectively. The collected cells were cultured in the above-mentioned bioreactor under different treatment conditions, i.e., different FeSO_4_·7H_2_O concentrations (0, 40, and 80 g/L), growth times (12, 36, and 50 h), and magnetic field intensities (0.05, 3.5, and 15 mT) with inoculation size of 0.01 g/L (Table S1). The corresponding treatment groups with different FeSO_4_·7H_2_O concentrations were Fe.0, Fe.40, and Fe.80, respectively. The groups treated with different times were Tim.12, Tim.36, and Tim.50m, and the groups treated with different magnetic fields were Fe.40 (i.e., Mag.0.05mT), Mag.3.5mT, and Mag.15mT. The FeSO_4_·7H_2_O in the 0K-FeSO_4_·7H_2_O media was replaced by 10 g/L sublimed sulfur to obtain 0 g/L FeSO_4_·7H_2_O group. The magnetic field was generated by placing hard ferrite magnets on the surface of the aerated bioreactor, and the 0.05-mT was the geomagnetic field intensity. The bacterial cells harvested from each experimental group were divided into four parts: transmission electron microscope (TEM) analysis, iron content measurement, transcriptome sequencing, and real-time quantitative PCR (RT-qPCR) analysis (Fig. 1).

### TEM observation and intracellular iron content determination

The first part of *A. ferrooxidans* BYM cells was fixed overnight with glutaraldehyde (2.5%, vol/vol) at 4°C, fixed with 1% (wt/vol) osmium tetroxide for 1 h, dehydrated through a graded ethanol series (70%, 80%, 90%, and 100%), and embedded in Epon 812 resin. UC7 ultramicrotome (Co. Leica Microsystems GmbH, Germany) was used to obtain ultrathin sections stained with both uranyl acetate and lead citrate and observed by the HITACHI H-7650 TEM (HITACHI, Tokyo, Japan) at a 120-kV accelerating voltage (23). Based on the TEM images, the number and size of Fe_3_O_4_ nanoparticles of all cells in the vision (at least 100 cells) were counted under different magnifications. The average number and size of Fe_3_O_4_ nanoparticles per cell were calculated.

The second part of the collected cells was placed in glass tubes and dried at 60°C until constant weight. The bacterial cells were treated with 0.5 mL HNO_3_ and HCl in the glass tube at 36°C and then digested in the water bath at 100°C until the liquid was clear. The total iron content of the cell digestion solution was measured by phenanthroline spectrophotometry (24). The intracellular iron content (μg/mg) was calculated with the equation m_1_/m_2_, where m_1_ and m_2_ are the total iron content (μg) and dry weight of bacterial cells (mg), respectively.

### RNA extraction and transcriptome sequencing

RNA of the third part bacterial cells was extracted using traditional TRIzol method followed by a DNase treatment step, and its amount was determined using NanoDrop 2000c Spectrophotometer (Thermo Fisher, MA, USA). RNA degradation and contamination were monitored on 1% agarose gels. The integrity and quality of RNA samples were evaluated by Agilent 4200 TapeStation (Agilent Technologies, CA, USA). The rRNA was removed by using Epicentre Ribo-Zero rRNA Removal Kit (Epicentre, WI, USA). The cDNA library was constructed by NEBNext Ultra II Directional RNA Library Prep Kit for Illumina (New England Biolabs, Beijing, China). The quantification was performed using Invitrogen Qubit 2.0 (Invitrogen, CA, MA) to dilute the library to 1.0 ng/µL after cDNA library construction. Agilent 2100 (Agilent Technologies, CA, USA) was used to determine the insert size of the library, and then the effective concentration of the library was quantified accurately by qPCR to ensure library quality. RNA-sequencing (RNA-Seq) libraries were prepared and sequenced on the Illumina Novogene (Novogene Company, Beijing, China) with a paired-end protocol (BGI, China).

### Quantification of reads and differential gene expression analyses

Fastp (v.0.19.7) was used to filter out low-quality reads (reads containing more than 20% bases with *Q*-value ≤ 10) to control the quality of raw reads. Clean reads were obtained and compared to the rRNA sequence with Bowtie2 (v.2.33). Hisat2 (v.2.1.0) was used to compare unmapped reads with the reference *A. ferrooxidans* BYM genome alignment (accession no. CP082238). The read count was obtained from the mapping results by RSEM (v.1.3.1). For analysis of DGEs, read counts were adjusted by edgeR (v.3.20.2) program. The *P* value is corrected by multiple hypothesis testing using Benjamini–Hochberg (false discovery rate) method, and the DEGs were detected (corrected *P* value < 0.05 and |log2 (fold_change) | ≥ 1). Hierarchical cluster analysis of differentially expressed gene (DEG) union was performed to assess the transcriptional pattern variations using Cluster (v.3.0). Venn diagrams were generated through Venny online freeware (v.2.0.2) to exhibit shared or specific DEGs among different pairwise comparisons. DEGs were screened out and classified by gene ontology (GO) functional significance enrichment analysis. The genomic, chemical, and systemic functional information was analyzed according to the KEGG database. KEGG pathway with a corrected *P* value < 0.05 was considered as significantly enriched by DEGs.

### Series test of clusters

Short Time-series Expression Miner software is a tool to analyze short time-series gene expression data. To obtain the detailed gene expression information of *A. ferrooxidans* BYM at different FeSO_4_·7H_2_O concentrations, growth times, and magnetic field intensities, the DEGs were clustered in the profiles based on gene expression patterns using the STEM. Clustered profiles with a *P* value ≤ 0.05 were defined as statistically significant. DEGs belonging to the same profile were anticipated to have similar patterns of expression. GO (Level 2) and KEGG (B_Class) functional enrichment analyses were performed for the genes in the significantly clustered profiles.

### Construction of co-expression modules based on WGCNA analysis

The gene co-expression network was performed using weighted gene coexpression network (WGCNA) package, which was installed from Bioconductor (http://bioconductor.org/biocLite.R). The Pearson coefficient was used to determine the similarity between two gene expression patterns. To obtain a scale-free network, the Pearson coefficients were weighted by a power function. Pearson coefficients between genes were used to construct a hierarchical clustering tree. Different branches of the hierarchical clustering tree represented different gene modules, and different colors represented different modules. Using Pearson coefficients, we calculated the correlation between the module feature genes and characters. Heat map was drawn according to the correlation coefficients. The genes in modules were analyzed, and the gene with the highest intramodular connectivity in each functional module was identified as a hub gene. The identified hub genes were further confirmed and visualized networks using Cytoscape (v.3.7.0).

### RT-qPCR verification of hub gene

Extracted total RNA from *A. ferrooxidans* BYM was reverse transcribed into the first-strand cDNA using FastKing gDNA Dispelling RT SuperMix (TIANGEN, Beijing, China). The primers of hub genes for qRT-PCR were designed using Primer BLAST (https://www.ncbi.nlm.nih.gov/tools/primer-blast/), and 16S rRNA gene was selected as the internal reference gene. All primers were synthesized by TSINGKE (Beijing, China) and are shown in Table S2. The qPCRs were conducted in LineGene 9600 Plus System (BIOER, Hangzhou, China) using 2 × T5 Fast qPCR Mix (SYBR Green I) (TSINGKE, Beijing, China) according to the program: 95°C for 1 min, 40 cycles at 95°C for 15 s, 60°C for 15 s, and 72°C for 30 s. Melting curve analyses were performed to check for the specificity of the amplifications. Relative expression values were determined by the ΔCt method (25).

### Prediction of TFs and gene regulatory network

The Predicted Prokaryotic Regulatory Proteins (P2RP) network service platform was used to predict the transcriptional factors (TFs) in *A. ferrooxidans* BYM genome. The interaction patterns between TFs and DEGs were predicted based on the STRING (version 11.0, https://string-db.org) online server, and all genes were functionally annotated using the COG database. The regulatory networks between TFs and DEGs in *A. ferrooxidans* BYM genome were visualized using Cytoscape 3.8.2 software.

### Statistical analyses

All experiments were performed in triplicate, and the results were expressed as mean ± standard deviation (SD). We applied the polynomial fitting method available in Origin 9.0 (OriginLab, Northampton, MA, USA) to fit the treatment condition factors (FeSO_4_·7H_2_O concentration, growth time, and magnetic field intensity) and Fe_3_O_4_ nanoparticle phenomic parameters (number, particle size, and intracellular iron content). The correlations between the Fe_3_O_4_ nanoparticle phenomic parameters and hub genes were analyzed by redundancy analysis of Canoco 5 (Microcomputer Power, Ithaca, NY, USA).

## RESULTS

### Effects on the number and size of Fe_3_O_4_ nanoparticles and intracellular iron content

**Fig 2 F2:**
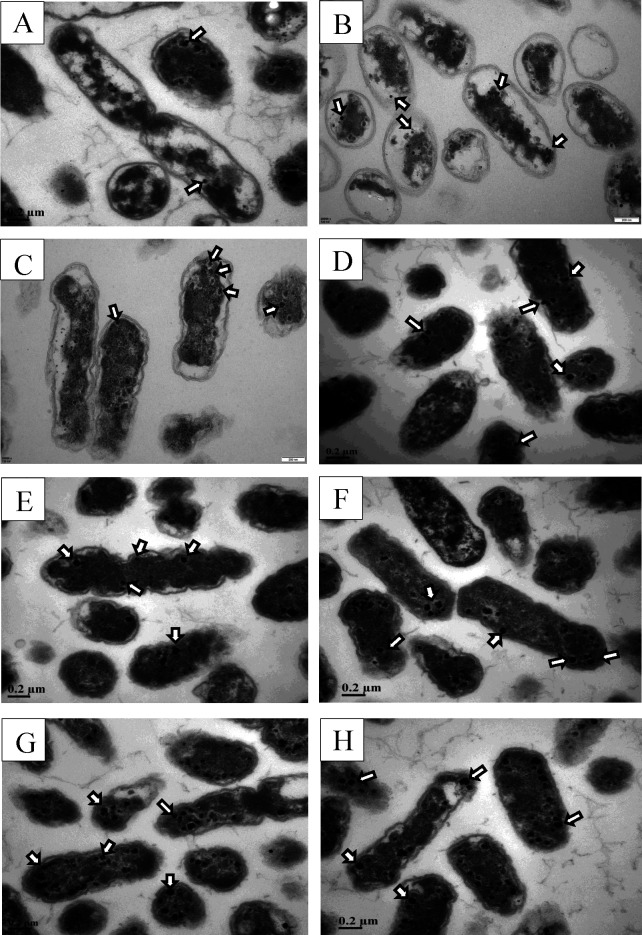
TEM observation of Fe_3_O_4_ nanoparticle in *A. ferrooxidans* BYM under different FeSO_4_·7H_2_O concentrations (A) 0, (B) 40, and (C) 80 g/L; growth times (D) 12, (E) 36, and (F) 50 h; and magnetic field intensities (G) 3.5 and (H) 15 mT.

TEM observations show that some Fe_3_O_4_ nanoparticles scattered in *A. ferrooxidans* BYM, and the average number and particle size changed under different treatments (Fig. 2). The average Fe_3_O_4_ nanoparticle number per cell harvested from the logarithmic phase (48 h) was 15.67 ± 2.08 (average ± SD) at 40g/L FeSO_4_·7H_2_O concentration and 0.05 mT magnetic field intensity. The average numbers of Fe_3_O_4_ nanoparticles decreased significantly (*P* < 0.01) when FeSO_4_·7H_2_O concentration changed to 0 or 80 g/L (Fig. 2A through C and 3A). Time course of the number of Fe_3_O_4_ nanoparticles in *A. ferrooxidans* cultured at 40g/L FeSO_4_·7H_2_O concentration and 0.05 mT magnetic field intensity was monitored. The results show that the average particle numbers per cell at 12, 36, and 50 h were 14.33 ± 1.53, 7.00 ± 1.00, and 15.33 ± 1.53, respectively (Fig. 2D through F and 3B). The average particle number per cell increased to a maximum value of 19.33 ± 2.52 and then decreased significantly (*P* < 0.01) with increasing magnetic field intensity from 0.05 to 15 mT [Fig F2 F3] Fig. 2 A, G, and H and 3C.

**Fig 3 F3:**
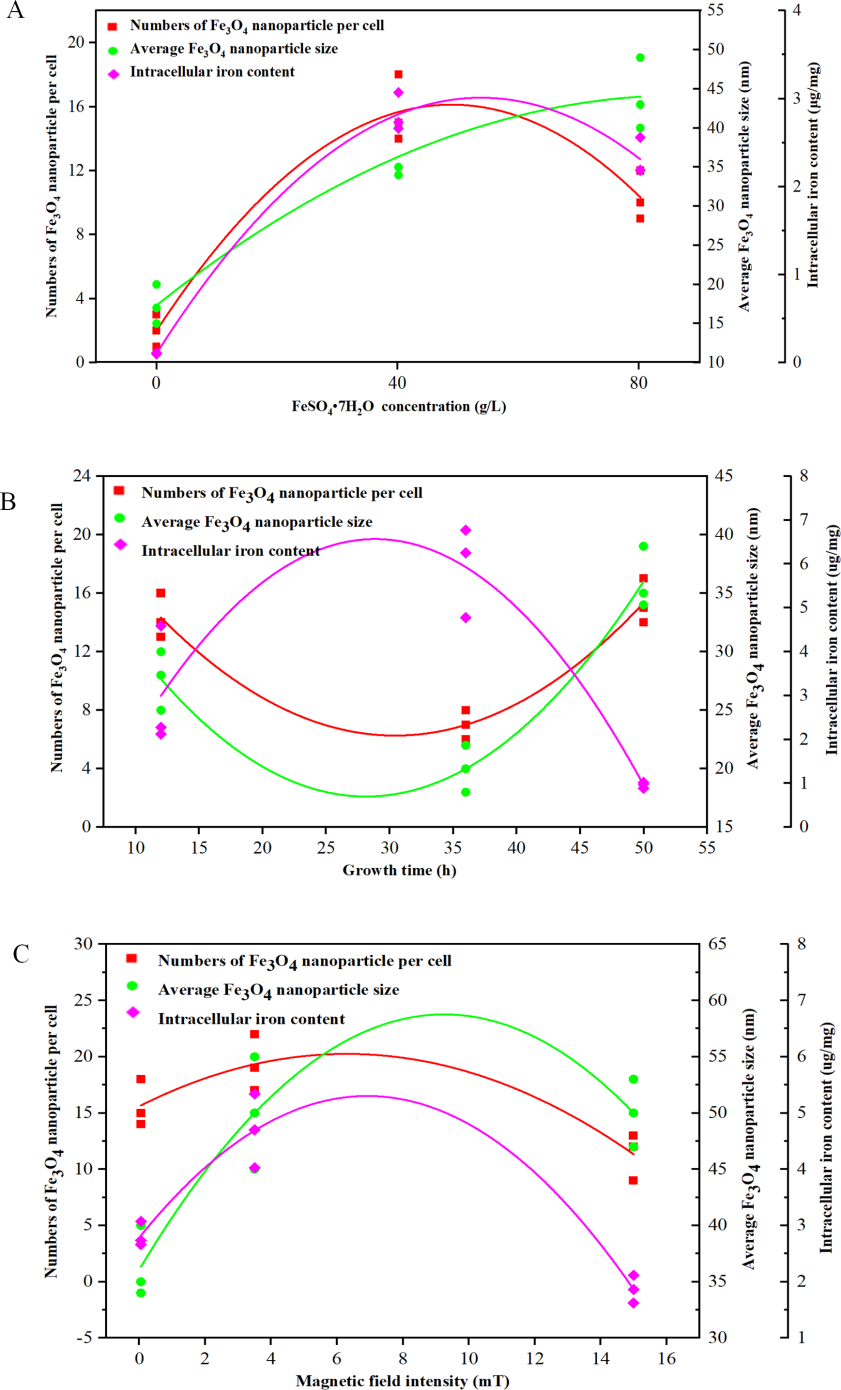
Effect of FeSO_4_·7H_2_O concentrations (**A**), growth times (**B**), and magnetic field intensities (**C**) on the Fe_3_O_4_ nanoparticle-related parameters (number, particle size, and intracellular iron content) and their relationship.

In addition, the average particle size of Fe_3_O_4_ nanoparticles changed significantly. The average particle size increased with the increase of FeSO_4_·7H_2_O concentration; the maximum particle size was 44.00 ± 4.58 nm (average ± SD) (Fig. 2A through C and 3A). It was also found that the average particle size varied during cell growth, and the minimum average particle size of Fe_3_O_4_ nanoparticle appeared at 36 h, which was 20.00 ± 2.00 nm (average ± SD) (Fig. 3B). The average particle size varied significantly when magnetic field intensity increased from 0.05 to 3.5 mT but almost unchanged when up to 15 mT (Fig. 2 A, G, and H and 3C).

After cell digestion, the intracellular iron content of *A. ferrooxidans* BYM was determined by phenanthroline spectrophotometry. It can be seen that the intracellular iron content sharply increased first and then significantly decreased with increasing FeSO_4_·7H_2_O concentration (Fig. 3A). Similar trends were observed under changes in growth time and magnetic field intensity (Fig. 3B and C). The maximum intracellular iron contents were 2.82 ± 0.22 µg/mg at 40g/L FeSO_4_·7H_2_O concentration, 5.93 ± 1.03 µg/mg at 36 h, and 4.69 ± 0.66 µg/mg at 3.5 mT magnetic field intensity, respectively. The regression analysis showed that the FeSO_4_·7H_2_O concentration, growth time, and magnetic field intensity were significantly correlated to the number and size of Fe_3_O_4_ nanoparticles and intracellular iron content (*R* ^2^ > 0.683, *P* < 0.01) (Table 1).

**TABLE 1:** Fitting function equation and related parameter values of different FeSO_4_·7H_2_O concentrations, growth times, magnetic field intensities, and Fe_3_O_4_ nanoparticle phenomic parameters

Treatment	Trait	Intercept	B1	B2	*R* ^2^	*P* value
FeSO_4_·7H_2_O concentration	Numbers of Fe_3_O_4_ nanoparticle per cell	2.000	0.579	5.94E−03	0.932	1.34E−04
	Average Fe_3_O_4_ nanoparticle size	17.333	0.617	3.54E−03	0.917	2.44E−04
	Intracellular iron content	0.105	0.108	1.01E−03	0.980	3.27E−06
Growth time	Numbers of Fe_3_O_4_ nanoparticle per cell	28.241	1.443	2.37E−02	0.889	5.84E−04
	Average Fe_3_O_4_ nanoparticle size	48.124	2.167	3.85E−02	0.890	5.67E−04
	Intracellular iron content	3.890	0.724	1.25E−02	0.816	2.61E−03
Magnetic field intensity	Numbers of Fe_3_O_4_ nanoparticle per cell	15.593	1.480	1.18E−01	0.683	1.34E−02
	Average Fe_3_O_4_ nanoparticle size	36.089	4.902	2.65E−01	0.744	7.06E−03
	Intracellular iron content	2.786	0.728	5.26E−02	0.893	5.17E−04
Equation		*y* = intercept + B1·*x* + B2·*x* ^2^				

### Overview of mRNA expression profiling

#### RNA-Seq and mapping

**Fig 4:**
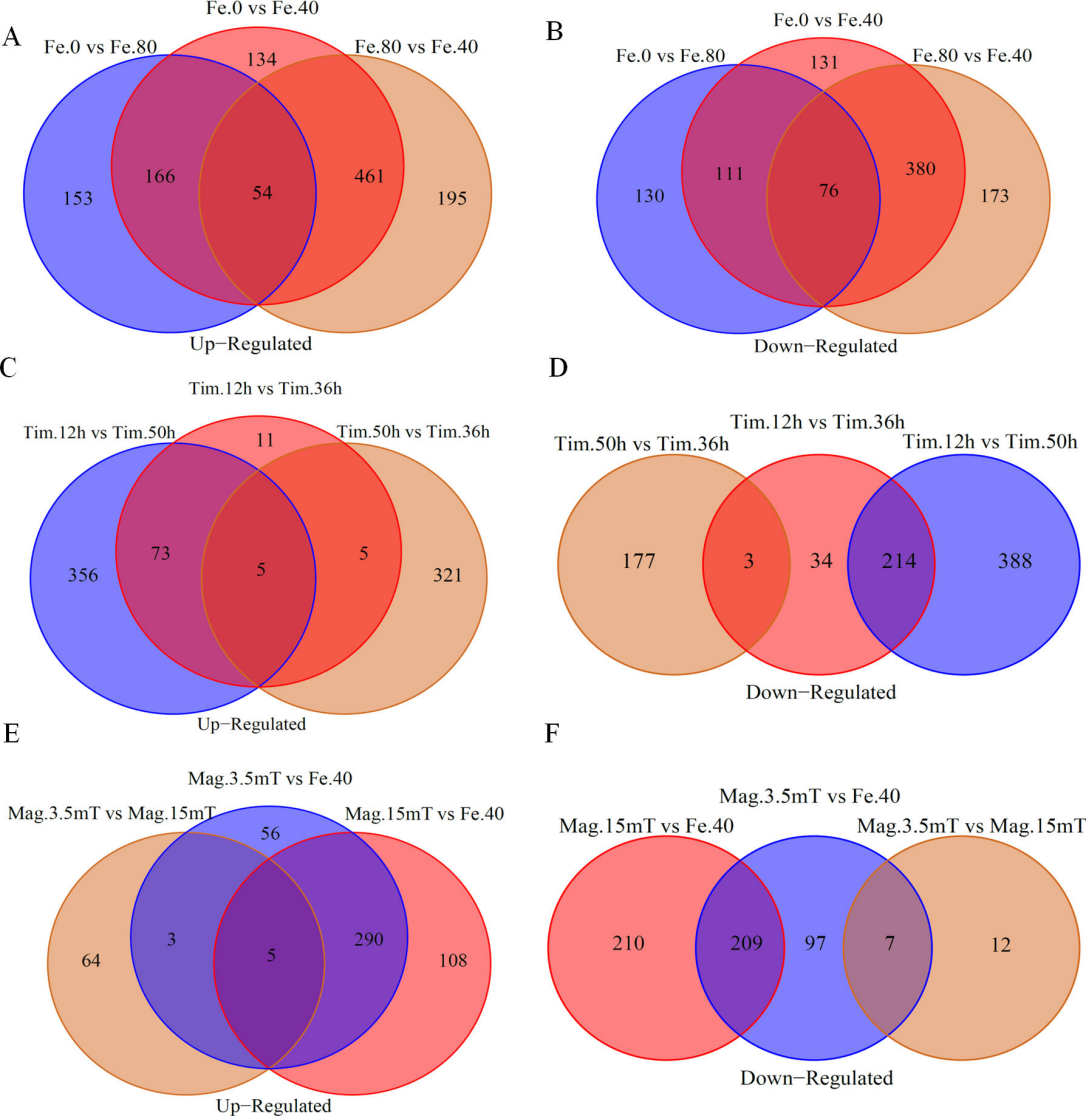
Venn diagram of up-regulated genes and down-regulated genes under different FeSO_4_·7H_2_O concentrations (**A and B**), growth times (**C and D**), and magnetic field intensities (**E and F**).

High-throughput RNA-Seq generated 57.34 Gb clean reads from 24 transcriptome samples. The data from each sample were greater than 1.22 Gb, and the Q20 and Q30 of each sample library was above 97.68% and 93.04%, respectively. Compared with the designated *A. ferrooxidans* BYM reference genome, the reads from each sample had alignment efficiency greater than 60%, except for Mag.3.5mT (Table S3). We conducted the BLAST of the unmatched sequences of Mag.3.5mT sample to other species; the results showed that the similarities were all lower than 1%, suggesting that the pollution rate of Mag.3.5mT sample is very low (Table S4). These results implied that the quality of the sequencing data was high and supported the validity of downstream analyses. The principal component analysis and the hierarchical clustering analysis were performed among replicates of each treatment; the results also indicated high levels of correlation and reproducibility within the same sample types (Fig. S1 and S2). Pairwise differential expression analysis between the treated samples showed that a large number of genes were significantly differentially expressed (*P* ≤ 0.05, |log2 fold change| > 0.6) under different FeSO_4_·7H_2_O concentrations, growth times, and magnetic field intensities (Fig. 4).

#### DEG analysis

As shown in the Venn diagram, the intergroup DEGs analysis was conducted. It can be found that Fe.0 vs Fe.40, Fe.80 vs Fe.40, and Fe.0 vs Fe.80 contained 1,513 (815 up- and 698 down-regulation), 1,339 (710 up- and 629 down-regulation), and 690 (373 up- and 317 down-regulation) DEGs, respectively. The change of FeSO_4_·7H_2_O concentrations resulted in 54 down-regulated DEGs and 76 up-regulated DEGs (Fig. 4A and B). There were 345 (94 up- and 251 down-regulation), 511 (331 up- and 180 down-regulation), and 1,036 (434 up- and 602 down-regulation) DEGs in Tim.12h vs Tim.36h, Tim.50h vs Tim.36h, and Tim.12h vs Tim.50h, respectively. Only five up-regulated DEGs existed in the three comparison groups, but there were no down-regulated DEGs. However, there were 214 down-regulated DEGs in Tim.12h vs Tim.36h and Tim.12h vs Tim.50h, but only three DEGs were down-regulated between Tim.12h vs Tim.36h and Tim.50h vs Tim.36h (Fig. 4C and D). Mag.3.5mT vs Mag.0.05mT (i.e., Fe.40), Mag.15mT vs Mag.0.05mT (i.e., Fe.40), and Mag.3.5mT vs Mag.15mT comprised 667 (354 up- and 313 down-regulation), 822 (403 up- and 419 down-regulation), and 91 (72 up- and 19 down-regulation) DEGs, respectively. There were only five up-regulated DEGs but no down-regulated DEGs in the three comparison groups (Fig. 4E and F).

#### Analysis of key GO terms and KEGG pathways

The BLAST2 GO tool was used to analyze the enriched functional GO terms, i.e., molecular function, biological process, and cellular component, associated with Fe_3_O_4_ nanoparticle synthesis in response to FeSO_4_·7H_2_O concentration. The GO functions of *A. ferrooxidans* BYM at different FeSO_4_·7H_2_O concentrations were mainly enriched in metabolic process, localization, cellular process, cellular anatomical entity, and catalytic activity, binding, etc. (Fig. S3A through C). The results of DEG functional enrichment of Fe.0 vs Fe.40 and Fe.40 vs Fe.80 were very similar; cells control the synthesis of Fe_3_O_4_ nanoparticles by regulating ion transport, membrane structure, and oxidation-reduction process, while the DEGs of Fe.0 vs Fe.80 were enriched in the metabolism of iron and sulfur (Fig. S3D through L). The GO functions of DEGsat different growth times and magnetic field intensities are consistent with the FeSO_4_·7H_2_O concentration treatment (Fig. S4A through C and S5A through C). The DEG expressions of metal ion absorption and transport, cell oxidation-reduction process, secondary metabolism, protein folding, and membrane structure function changed with the extension of growth time and the increasing magnetic field intensity (Fig. S4D through L and S5D through L). The main enrichment result of KEGG metabolic pathway in different conditions (i.e., FeSO_4_·7H_2_O concentrations, growth times, and magnetic field intensities) are consistent with the GO enrichment results (Fig. S6 through S8).

### Gene expression profiles of *A. ferrooxidans* BYM under FeSO_4_·7H_2_O concentration, growth time, and magnetic field intensity treatments

In order to study the regulatory mechanism of *A. ferrooxidans* BYM under different treatments, the series test of cluster analysis was used to obtain significant trend profiles. Genes with the same trend were clustered, 24 profiles with statistical significance were obtained, and eight colored profiles have a statistically significant number of genes assigned (*P* < 0.01) (Fig. S9). The GO enrichment results of the genes in eight colored profiles were similar. Metabolic and cellular processes, cellular anatomical entity, catalytic activity, and binding were found to be the most significant enrichments (Fig. S10A and B, S11A through C, and S12A through C). For the treatments with different concentrations of FeSO_4_·7H_2_O, the genes in Profile 2 were gathered into different KEGG pathways, including translation, metabolism of cofactors and vitamins, energy metabolism, carbohydrate metabolism, and amino acid metabolism (Fig. S10C), while the genes screened in Profile 1 were enriched in signal transduction and energy metabolism (Fig. S10D). At different growth times, the KEGG enrichment results showed that Profile 0 was significantly enriched in translation, metabolism of cofactors and vitamins, energy metabolism, carbohydrate metabolism, and amino acid metabolism (Fig. S11D). In Profile 7, signal transduction, energy metabolism, and carbohydrate metabolism were significantly enriched (Fig. S11E). Profile 3 was significantly enriched for energy metabolism and amino acid metabolism (Fig. S11F). Under different magnetic field intensities, the enrichment analysis of KEGG pathway showed that Profile 1 was significantly enriched for signal transduction, metabolism of cofactors and vitamins, energy metabolism, carbohydrate metabolism, and amino acid metabolism (Fig. S12D). Profile 0 was enriched for signal transduction, membrane transport, energy metabolism, and cellular community prokaryotes (biofilm formation and quorum sensing) (Fig. S12E). Profile 6 significantly enriched metabolic pathways, including metabolism of cofactors and vitamins; folding, sorting, and degradation; energy metabolism; and amino acid metabolism (Fig. S12F).

### Predictive construction of gene regulatory networks for Fe_3_O_4_ nanoparticle synthesis in *A. ferrooxidans* BYM

TFs play a key role in gene transcription and regulation. One hundred unique putative TFs were identified from the *A. ferrooxidans* BYM genome, among which 29 TFs cooperated (Table S5; Fig. S13). Cytoscape software was utilized to construct a regulation network of the DEGs and TFs (Fig. S14). Among 19 TFs involved in the regulation network, 12 TFs were involved in transcription, two TFs were involved in inorganic ion transport and metabolism and signal transduction mechanisms, and the remaining three TFs participated in function unknown. TFs and DEGs interact together to form the gene regulation network of *A. ferrooxidans* BYM, including the gene regulation network of Fe_3_O_4_ nanoparticle synthesis. DEGs in the regulatory network were found to be involved in energy production and conversion, coenzyme transport and metabolism, amino acid transport and metabolism, carbohydrate transport and metabolism, lipid transport and metabolism, translation, ribosomal structure and biogenesis, transcription, etc. (Fig. S14).

### Gene coexpression network construction and visualization

**Fig 5:**
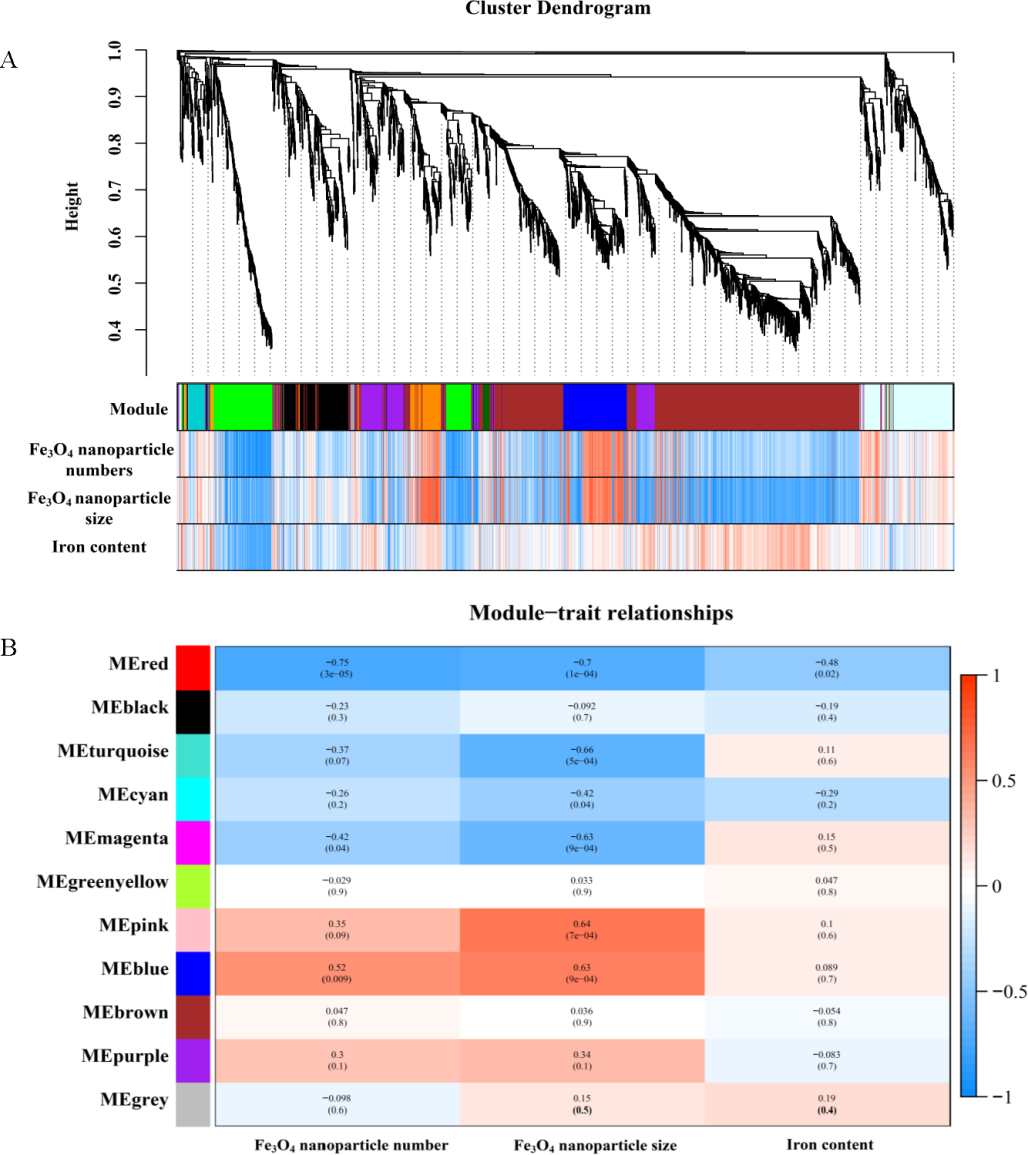
Gene dendrogram with trait (**A**) and module trait correlation (**B**) based on WGCNA co-expression network.

**Fig 6:**
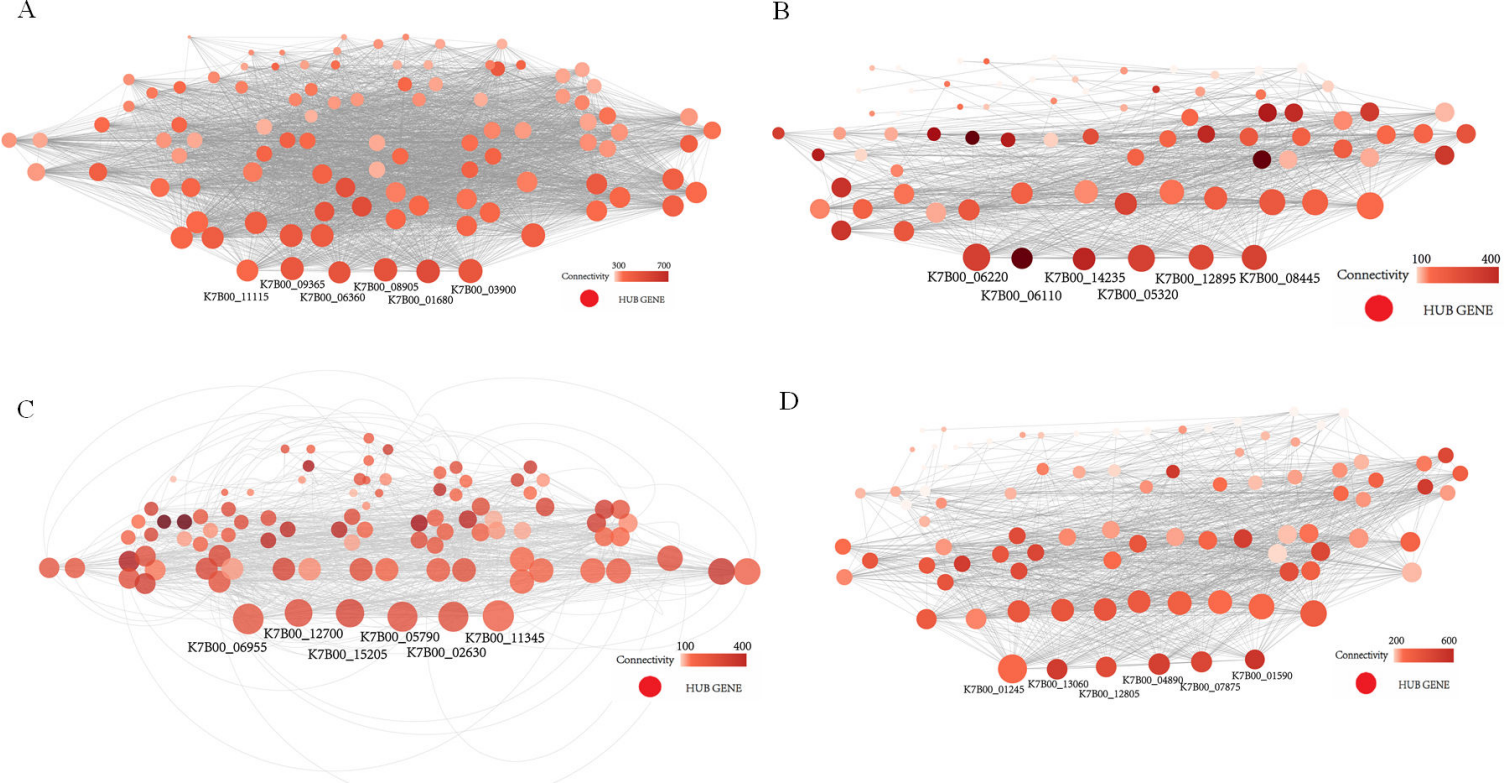
The correlation network of MEblue (**A**), MEpink (**B**), MEred (**C**), and MEmagenta (**D**) modules.

Clustering results from 24 samples showed that the gene expression of Fe.80 and Fe.40, Mag.15mT, and Mag.3.5mT was similar (Fig. S15). Modules with similar expressions were then combined by the dynamic shear tree method to obtain the co-expressed gene modules (Fig. 5A). The expression profile of each cluster eigengene was shown as a heat map, and the eigengenes were clustered into 11 correlated modules (Fig. 5B). MEBlue, MEPink, MERed, and MEMagenta had the highest correlations with Fe_3_O_4_ nanoparticle phenomic parameters among these modules. Among them, MEblue and MEpink were positively correlated with both the number and size of Fe_3_O_4_ nanoparticles, while MEred and MEmagenta appeared to have a negative correlation. Therefore, 24 eigengenes possibly related to iron metabolism in the four models were finally screened (Fig. 6).

### Correlation analysis of hub genes with the Fe_3_O_4_ nanoparticle phenomic parameters

**Fig 7:**
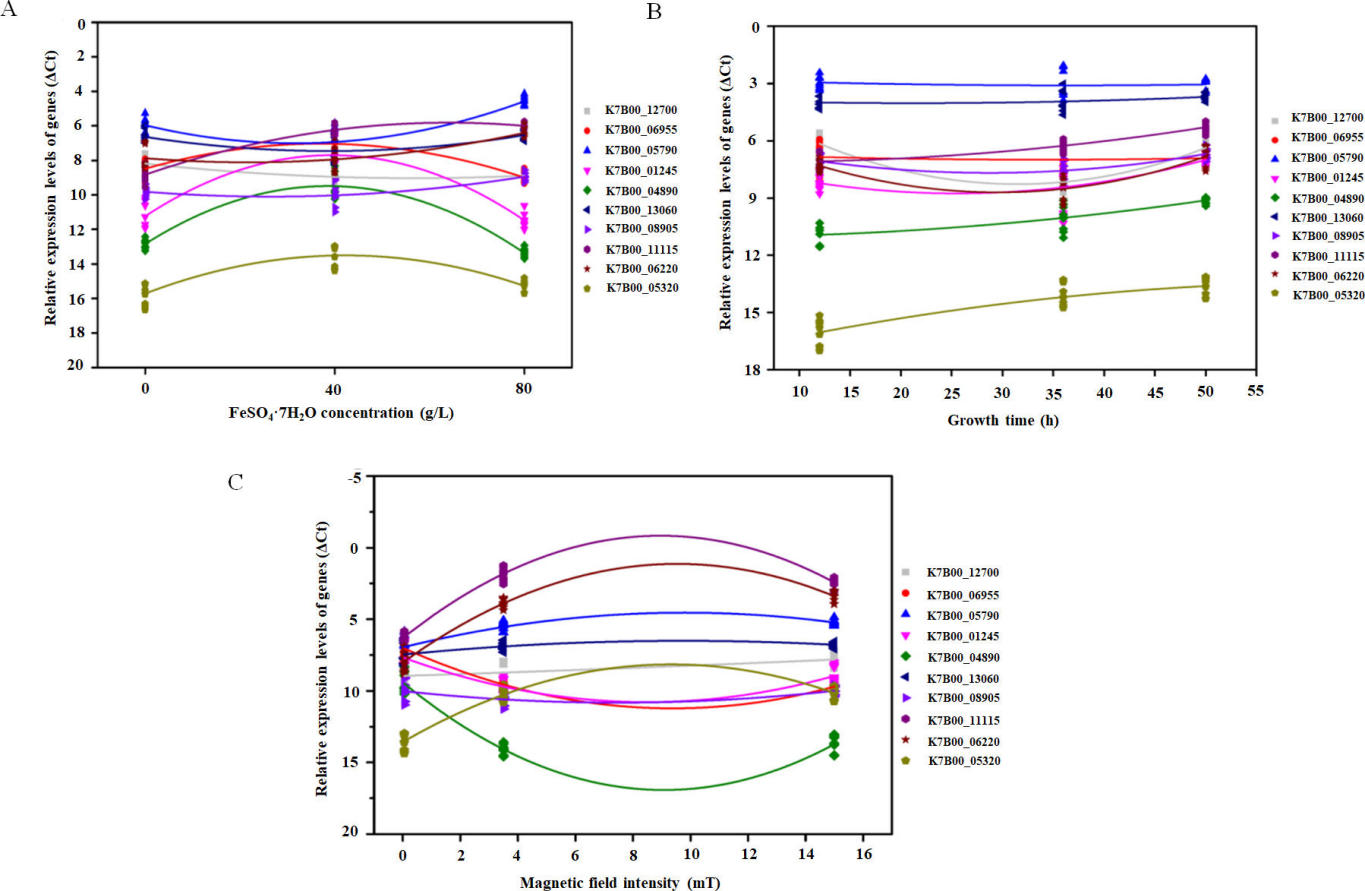
Correlations between gene expression level with FeSO_4_·7H_2_O concentrations (**A**), or growth time (**B**), or magnetic field intensities (**C**).

According to further analysis of the function of 24 eigengenes, 10 hub genes related to the formation of Fe_3_O_4_ nanoparticles were finally selected as analytical objects (Table 2). The qRT-PCR validations for selected genes of interest were consistent with RNA-Seq data. Fig. 7 shows the multivariate fitting analysis results of the relative expression levels of 10 hub genes under different treatments. With the increase of FeSO_4_·7H_2_O concentration, the expression levels of K7B00_06955, K7B00_01245, K7B00_04890, and K7B00_05320 were firstly increased and then decreased, while the opposite trends were observed for K7B00_05790, K7B00_06220, and K7B00_08905. FeSO_4_·7H_2_O concentration was significantly correlated with the expression levels of these genes (*R* ^2^ > 0.500, *P* < 0.01) (Fig. 7A; Table 3). In addition, the relative expression levels of K7B00_05790, K7B00_13060, and K7B00_06955 were little affected by growth time. There was no significant correlation between growth time and the expression levels of these genes (*R* ^2^ < 0.048, *P* > 0.05). In contrast, the relative expression levels of K7B00_12700, K7B00_06220, and K7B00_01245 decreased first and then increased with the extension of growth time, which was significantly correlated with the growth time (*R* ^2^ > 0.404, *P* < 0.01) (Fig. 7B; Table 3).

**TABLE 2:** Functional annotation for 24 eigengenes

Gene	Source	Length (bp)	Functional annotation
K7B00_12700	MEred	720	HdrC protein
K7B00_06955		1,377	MntH protein
K7B00_05790		462	Heme-binding sites
K7B00_15205		1,035	*N*-Acetyl-gamma-glutamyl-phosphate reductase
K7B00_02630		450	Rhodanese-like domain-containing protein
K7B00_11345		2,478	DUF2309 domain-containing protein
K7B00_01245	MEmagenta	1,014	Ferrochelatase
K7B00_04890		1,386	Voltage-gated ion channels
K7B00_13060		1,263	NADH-quinone oxidoreductase subunit NuoF
K7B00_12805		801	YdcF family protein
K7B00_07875		837	Pantoate-beta-alanine ligase
K7B00_01590		780	Iron export ABC transporter permease subunit
K7B00_08905	MEblue	828	TonB family protein
K7B00_11115		1,110	Hem-binding sites
K7B00_09365		693	Glycosyltransferase
K7B00_01680		1,128	FAD-dependent oxidoreductase
K7B00_03900		801	DUF5666 domain-containing protein
K7B00_06360		1,344	Aminomethyl-transferring glycine dehydrogenase subunit GcvPA
K7B00_06220	MEpink	204	Cytochrome bd oxidase small subunit
K7B00_05320		1,881	Voltage-gated ion channels
K7B00_06110		1,008	tRNA glutamyl-Q(34) synthetase GluQRS
K7B00_14235		423	Glycine cleavage system protein H
K7B00_12895		336	1,6-Anhydro-*N*-acetylmuramyl-L-alanine amidase AmpD
K7B00_08445		1,389	Dihydrolipoyl dehydrogenase

**TABLE 3:** Fitting function equation and related parameter values of different FeSO_4_·7H_2_O concentrations, growth times, magnetic field intensities, and hub gene expression

Treatment	Gene	Intercept	B1	B2	*R* ^2^	*P* value
FeSO_4_·7H_2_O concentration	K7B00_12700	8.182	0.030	2.63E−04	0.351	2.13E−03
	K7B00_06955	8.487	0.079	1.06E−03	0.738	3.92E−08
	K7B00_05790	5.958	0.066	1.04E−03	0.753	1.97E−08
	K7B00_01245	11.239	0.180	2.29E−03	0.866	1.28E−11
	K7B00_04890	12.848	0.174	2.26E−03	0.939	8.88E−16
	K7B00_13060	6.636	0.042	5.44E−04	0.387	1.09E−03
	K7B00_08905	9.813	0.021	4.05E−04	0.509	7.57E−05
	K7B00_11115	8.830	0.094	7.37E−04	0.944	3.33E−16
	K7B00_06220	7.872	0.022	5.04E−04	0.594	7.67E−06
	K7B00_05320	15.721	0.105	1.24E−03	0.760	1.37E−08
Growth time	K7B00_12700	2.753	0.350	5.56E−03	0.794	2.26E−09
	K7B00_06955	6.647	0.020	3.03E−04	0.073	8.92E−01
	K7B00_05790	2.753	0.019	2.62E−04	0.061	7.83E−01
	K7B00_01245	6.852	0.149	2.93E−03	0.404	7.74E−04
	K7B00_04890	11.049	0.001	7.48E−04	0.697	2.25E−07
	K7B00_13060	3.852	0.017	3.95E−04	0.048	2.11E−01
	K7B00_08905	5.870	0.126	2.20E−03	0.445	3.30E−04
	K7B00_11115	7.079	0.011	9.31E−04	0.830	2.25E−10
	K7B00_06220	4.757	0.267	4.51E−03	0.676	5.12E−07
	K7B00_05320	17.377	0.123	9.52E−04	0.767	9.82E−09
Magnetic field intensity	K7B00_12700	8.958	0.064	8.06E−04	0.446	3.17E−04
	K7B00_06955	6.996	0.901	4.80E−02	0.883	2.54E−12
	K7B00_05790	6.959	0.494	2.52E−02	0.663	8.12E−07
	K7B00_01245	7.661	0.735	4.33E−02	0.592	8.14E−06
	K7B00_04890	9.409	1.655	9.11E−02	0.933	3.33E−15
	K7B00_13060	7.465	0.197	1.01E−02	0.252	1.18E−02
	K7B00_08905	10.015	0.216	1.45E−02	0.194	2.89E−02
	K7B00_11115	6.315	1.597	8.92E−02	0.972	0.00E + 00
	K7B00_06220	8.015	1.443	7.56E−02	0.949	1.11E−16
	K7B00_05320	13.569	1.161	6.22E−02	0.907	1.68E−13
Equation		*y* = intercept + B1·*x* + B2·*x* ^2^				

With increasing magnetic field intensity, the expression levels of K7B00_11115, K7B00_06220, K7B00_05790, and K7B00_05320 all increased first and then decreased and were significantly correlated with magnetic field intensity (*R* ^2^ > 0.663, *P* < 0.01). In addition, the expression levels of K7B00_06955, K7B00_01245, and K7B00_04890 decreased first and then increased with the increase of magnetic field intensity and were significantly correlated with magnetic field intensity (*R* ^2^ > 0.592, *P* < 0.01). The relative expression levels of K7B00_13060 and K7B00_08905 were seldom influenced by magnetic field intensity, implying that magnetic field intensity was not significantly correlated with the expression levels of these two genes (*R* ^2^ < 0.252, *P* > 0.01) (Fig. 7C; Table 3). The expression levels of 10 hub genes were significantly correlated with the number and size of Fe_3_O_4_ nanoparticles but were less correlated with intracellular iron content (Fig. S16).

## DISCUSSION

### Response of Fe_3_O_4_ nanoparticle phenomic parameters to environmental conditions

As an acidophilic chemolithoautotroph, *A. ferrooxidans* can obtain energy from the oxidation of Fe(II) or reduced sulfur for growth (26). It has been proven that *A. ferrooxidans* incubated with FeSO_4_·7H_2_O can produce Fe_3_O_4_ nanoparticle, i.e., magnetosome, which is also synthesized by MTB and exhibits potential in medical and biotechnological applications (27). Magnetosome synthesis in MTB seems to positively respond to the changes in culture conditions, such as FeSO_4_·7H_2_O concentration, growth time, and magnetic field intensity (28). Our study also indicated that FeSO_4_·7H_2_O concentration significantly affects Fe_3_O_4_ nanoparticle synthesis in *A. ferrooxidans* (Table 1). In the present study, 40 g/L FeSO_4_·7H_2_O was appropriate for synthesizing Fe_3_O_4_ nanoparticles with more numbers and bigger particle sizes (Fig. 2B and 3A). Once FeSO_4_·7H_2_O concentration increased to 80 g/L, the synthesis of Fe_3_O_4_ nanoparticle was inhibited, but the particle size showed no significant effect (Fig. 2C and 3A). It was found that FeSO_4_·7H_2_O concentration had similar effects on the intracellular iron content of *A. ferrooxidans* BYM (Fig. 3A). These results indicated that too high iron concentration would inhibit the formation of Fe_3_O_4_ nanoparticles, consisting with the results were shown in MTB (29, 30). However, Fe_3_O_4_ nanoparticles were still observed in the TEM of *A. ferrooxidans* BYM incubated with sublimed sulfur (Fig. 2A). This abnormal situation might be attributed to the presence of residual nanoparticles, which were inherited from the parent cells cultured with FeSO_4_·7H_2_O but could be eliminated by increasing the passage number of *A. ferrooxidans* cultivated with reduced sulfur (31).

The magnetosomes exhibit differences in size and stoichiometry due to their formation depending on the bacterial growth phase (21). It has been reported that some small magnetosome crystals began to produce in *M. magneticum* AMB-1 after 24 h culture, then grew gradually at 48 h, and matured at 72 h (32). The key time point for cell growth and magnetosome formation of *M. gryphiswaldense* MSR-1 was found to be 18–20 h. At this period, cells entered the log phase of growth and a high quantity of magnetosome (26.00 ± 3.00 per cell) was produced (33). Accordingly, the cell growth time significantly and directly affects the formation of Fe_3_O_4_ nanoparticles (Table 1). Time-course monitoring of Fe_3_O_4_ nanoparticle formation during the growth of *A. ferrooxidans* BYM shows that the number and particle size first decreased and then increased over time (Fig. 2D through F and 3B). The nanoparticles observed at an early stage may have been the magnetosomes in cells inherited from the original inoculum (21). For *M. gryphiswaldense* MSR-1, there were two mechanisms to distribute magnetosomes to daughter cells. During replication, one daughter cell might contain the majority of magnetosomes while another daughter cell might comprise very few or no magnetosomes, or each daughter cell might receive exactly half the number of magnetosomes from the mother cell (34). This provided a heuristic for explaining the small number of Fe_3_O_4_ nanoparticles in *A. ferrooxidans* BYM at 36 h (Fig. 3B). The cells divided vigorously to produce new daughter cells at 36 h, fewer Fe_3_O_4_ nanoparticles were allocated to the daughter cells, and new Fe_3_O_4_ nanoparticles with small particle sizes further formed in these new cells. The matured Fe_3_O_4_ nanoparticles would remain stable in the cells until they were buried and fossilized in nature (35). In addition, the maximum intracellular iron content occurred at 36 h, but the number and size of Fe_3_O_4_ nanoparticles were not maximal at this time point (Fig. 3B). It has been reported that the magnetosome yields did not keep increasing despite intracellular iron content continuously increasing (36). Therefore, intracellular iron content did not directly affect the synthesis of magnetosomes in *A. ferrooxidans* BYM.

The Earth has a constant geomagnetic field that creates relatively stable environmental conditions (37). MTB exhibits the capability of sensing magnetic field to navigate and move along geomagnetic field lines due to the presence of magnetosomes (4). It has been proven that *A. ferrooxidans* BYM synthesizes a small amount of Fe_3_O_4_ nanoparticles scattered in the cell and appears weak magnetotaxis (9). Low magnetic field intensity with a range from 0.05 to 3.5 mT could promote the synthesis of Fe_3_O_4_ nanoparticle and iron uptake, which descended once the strong magnetic field intensity over 3.5 mT and the minimum values occurred at 15 mT and Fig. 2 A, G, and H and 3C. However, it has been documented that strong magnetic field intensity (0.2 T) promoted the formation of magnetosomes in *M. magneticum* AMB-1 (16). The contradictory trends might be due to growing and magnetically responding dissimilarity between *A. ferrooxidans* and MTB (18). Magnetic field intensity was significantly correlated to the formation of Fe_3_O_4_ nanoparticles in *A. ferrooxidans* BYM (Table 1). The magnetic fields can alter the structure of microbial cell membranes and DNA by generating transmembrane potential and Lorentz force, resulting in the change of biological behavior (38). Thus, the interaction between the magnetic field and Fe_3_O_4_ nanoparticles in *A. ferrooxidans* BYM created mechanical forces, which could affect ion channels or damage the cell membrane and further change cell status.

### Response of important biological processes to environmental conditions

Key biological processes and metabolic pathways of *A. ferrooxidans* BYM in response to FeSO_4_·7H_2_O concentration, growth time, and magnetic field intensity could be found through meta-analysis of large RNA-Seq data sets (39). The GO functional enrichment results of DEGs in Fe.0 vs Fe.40 and Fe.80 vs Fe.40 showed that the functions of the identified DEGs were mainly oxidation-reduction process, ion transport, cell structure, etc. (Fig. S3). It has been reported that the formation of magnetic nanoparticles in *A. ferrooxidans* and MTB involved the uptake and transport of iron, the change of membrane structure, and oxidation-reduction process (40). The genome of *A. ferrooxidans* ATCC 23270 contains several open reading frames with high similarity to magnetosome genes of MTB, such as *mpsA*, *mamE*, *mamB*, and *magA*, which are associated with magnetosome membrane formation, oxidation-reduction process, iron transport, and iron uptake, respectively (23). It was also found that the expression levels of *mpsA-*, *thy*-, *magA*-, and *mamB*-like genes in *A. ferrooxidans* ATCC 23270 decreased with the increase of FeSO_4_·7H_2_O concentration (41). The above results indicated that FeSO_4_·7H_2_O concentration might affect the formation of Fe_3_O_4_ nanoparticles in *A. ferrooxidans* BYM by influencing the formation of magnetosome membranes, iron oxidation-reduction process, uptake, and transport of iron. Notably, Fe.0 vs Fe.80 shared few DEGs, which were mainly enriched in iron and sulfur metabolism by GO annotation (Fig. 4A and B; Fig. S3). There is a lack of iron for the synthesis of Fe_3_O_4_ nanoparticles in *A. ferrooxidans* when utilizing elemental sulfur as the sole energy source (27). Additionally, high concentration of iron poses severe oxidative stress to living cells, which could cause cells to mainly focus on autogenous growth and iron metabolism (42). These results showed that the change of energy source for *A. ferrooxidans* BYM led to the significant expression of metabolism-associated genes, rather than the expression of genes regulating Fe_3_O_4_ nanoparticle synthesis. The DEG expression trend in Profile 2 also presents a similar pattern (Fig. S9A and S10). The energy substrate has been reported to affect microbial growth and metabolic activity directly (43). The key biological processes and metabolic pathways of DEGs in Profile 1 indicated that *A. ferrooxidans* adopted different regulatory mechanisms to deal with the different energy substrates (Fig. S9A and S10). The KEGG pathway mainly enriched important biochemical processes, such as ribosome and oxidative phosphorylation (Fig. S6). The ribosome synthesis ability increased in the face of environmental stresses, which might somewhat improve the translation function (44). It has been documented that oxidative phosphorylation is the final metabolic pathway of cellular respiration and a key step in ATP production (45). These implied that FeSO_4_·7H_2_O concentration affected the synthesis of Fe_3_O_4_ nanoparticles by altering the normal growth and metabolism of *A. ferrooxidans* BYM.

The samples of *A. ferrooxidans* BYM at different growth times were analyzed to harvest DEGs due to the sequential expression of genes associated with the magnetosome synthesis in some strains of MTB (21). More DEGs enriched in Tim.12h vs Tim.50h were annotated to oxidation-reduction process, ion transmembrane transport, plasma membrane, and periplasmic space change based on GO term analysis (Fig. 4C and D; Fig. S4). The previous study showed that *A. ferrooxidans* grew to a logarithmic stage rapidly after 48 h (23). At this time, more Fe_3_O_4_ nanoparticles accumulated in the cell (Fig. 2). It has also been found that *M. gryphiswaldense* MSR-1 produces a large number of magnetosomes at the end of the logarithmic phase (46). The difference of Fe_3_O_4_ nanoparticle production capacity in different growth stages of *A. ferrooxidans* BYM may be one of the reasons for the higher DEGs of Tim.12h vs Tim.50h. In addition, protein synthesis and ribosome production account for most nutrient and energy consumption in a fast-growing cell (47). Therefore, the metabolic pathways of the ribosome and oxidative phosphorylation were primarily organized in KEGG pathways of the DEGs in Tim.12h vs Tim.50h (Fig. S7). Besides, KEGG analysis of gene expression trends for the samples at different growth times showed that the significantly enriched biological functions were related to cell energy metabolism and signal transduction (Fig. S9C and D and S11). The signal transduction system might allow bacteria to adapt to various adverse environments (48). The presence of extraordinarily complex signal transduction pathways in MTB might reflect their adaptation to complex chemical gradients in environments (49). Fe_3_O_4_ nanoparticles gradually synthesized in *A. ferrooxidans* BYM accompanying enhancement of cell signal transductions with the extension of growth time, suggesting their possibly certain role in intercellular signaling to cope with environmental change.

Magnetic fields affect not only ferromagnetic materials but also paramagnets like oxygen, sodium, DNA, proteins, and even water molecules, all of which are important for regulating cellular processes (37). The lowest number of co-expressed DEGs in Mag.3.5mT vs Mag.15mT (Fig. 4E and F) showed that the transcriptomic response of *A. ferrooxidans* BYM to the geomagnetic field and external magnetic field was different. In addition, the gene expression level in Profile 1 and Profile 6 also indicated that the external magnetic field affected the expression of some genes related to cell metabolism (Fig. S9C and S12). It has been shown that MTB can respond to the applied external magnetic field, which might promote magnetosome synthesis (49). The DEGs of *A. ferrooxidans* BYM under the magnetic field with different intensities by GO functional enrichment showed that the component of the membrane was significantly enriched (Fig. S5). It is worth noting that the plasma membrane strongly responds to the change of magnetic field intensity, which is the primary site of interaction between biological systems and static magnetic fields (50, 51). Therefore, the magnetic field intensity might affect the synthesis of Fe_3_O_4_ nanoparticles in *A. ferrooxidans* by controlling membrane formation. On the basis of the KEGG pathway analysis, we noted that ABC transporters and quorum sensing metabolic pathways were significantly enriched with increasing magnetic field intensity (Fig. S8 and S12). Expression of the metabolic pathway of ABC transporters indicated that the cell required energy, carbohydrates, and amino acids to repair damage caused by magnetic field treatment (48). Quorum sensing is a bacterial communication system that has the ability to continuously secrete signal molecules to the outside during reproduction (9). It regulates a number of biological characteristics, such as motility, biofilm formation, and colonization, which are necessary for the survival of bacteria (52). It has been proven that a strong magnetic field appears to have a positive effect on some biological characteristics, i.e., bioluminescence, while others attest to a negative effect on the self-regulating function of bacteria (48). *A. ferrooxidans* can adapt to the environment through chemotactic movement and quorum sensing (9). When the appropriate magnetic field is introduced, the positive chemotaxis of the quorum sensing leads to an increase in cell density, resulting in an increase in magnetosome yield. Once the magnetic field is too strong, the destruction of quorum sensing by magnetic field would hinder the self-regulating function of cells and might destroy its ability to regulate physiological activities. Therefore, *A. ferrooxidans* might accommodate the magnetic field intensity change by increasing the expression of the quorum sensing system in order to survive, promoting the generation of Fe_3_O_4_ nanoparticles.

### Inference of regulatory network of genes involved in Fe_3_O_4_ nanoparticle synthesis

Gene regulatory networks play an essential role in controlling gene expression and enabling the organism to function properly (53). Although some molecular studies have been performed in Fe_3_O_4_ nanoparticle synthesis in *A. ferrooxidans*, a comprehensive insight into its transcriptional regulatory network is still lacking. Integrating TFs with the DEGs to construct a transcriptional regulatory network model and to predict putative causal regulations between them would help us understand the synthesis mechanism of Fe_3_O_4_ nanoparticles in *A. ferrooxidans* BYM. TFs are the main operators of transcriptional reprogramming under abiotic stress (54). Twenty-nine TFs in the genome of *A. ferrooxidans* BYM cooperated with each other to regulate cell growth and metabolism (Table S5; Fig. S13). Among them, K7B00_14970 (Sigma factors, RpoN) and K7B00_10240 (TF, Trans_reg_C) were associated with 27 other TFs. Sigma factors are necessary for controlling transcription and regulating a wide range of genes associated with diverse functions. They can affect many critical cellular activities, such as growth, stress tolerance, initiative biofilm formation, and adaptation to environmental changes (55). The trans_reg_C structure exhibits DNA-binding function and is only present in prokaryotic two-component systems, which are widely used signal transduction devices in bacteria and participate in a variety of gene regulatory systems in response to changes in growth conditions (56, 57). Therefore, K7B00_14970 and K7B00_10240 played key roles in the gene regulatory network of *A. ferrooxidans* BYM. Notably, the biological processes involved in the transcriptional regulatory network based on TFs and DEGs mainly included transcriptional regulation, energy generation and transformation, inorganic ion transport and metabolism, cell wall/membrane/capsule biosynthesis, etc. (Fig. S14). This was consistent with the functional enrichment results of DEGs and profiles (Fig. S3 to S14). To some extent, these findings indicate that the predicted gene regulatory network for Fe_3_O_4_ nanoparticle synthesis is referential. Therefore, the Fe_3_O_4_ nanoparticle synthesis of *A. ferrooxidans* BYM seems to involve the processes of membrane formation, iron transportation and redox control, etc. (40). However, more data are needed to explain the accurate process of Fe_3_O_4_ nanoparticle synthesis in *A. ferrooxidans* BYM.

### Hypothetical molecular model of Fe_3_O_4_ nanoparticle synthesis based on hub regulatory genes

WGCNA can correlate modules with external sample traits to find eigengene modules or an intramodular hub gene (58). A total of 24 eigengenes related to iron metabolism were selected (Table 2; Fig. 6). Most of them are involved in cellular transcription regulation, oxidation–reduction process, ion transport, and membrane structure, which is consistent with the results of important biological processes in response to environmental conditions. On the basis of the NCBI sequence alignment results, we finally identified 10 hub genes related to the synthesis of Fe_3_O_4_ nanoparticles, which were significant expressions under different treatment conditions (Table 2; Fig. 7). The Fe_3_O_4_ nanoparticle synthesis in *A. ferrooxidans* requires a large amount of iron, so the uptake and transport of Fe(II)/Fe(III) are the most critical process (40). K7B00_08905 encoding TonB-dependent ferric transport, and the K7B00_06955 encoding Fe(II) NRAMP transporter (MntH), might closely perform the function of Fe(III) and Fe(II) transport (59, 60). In addition, the K7B00_05320 and K7B00_04890 encode voltage-gated ion channels belonging to transmembrane proteins. Cell membranes are usually impermeable to ions, which must diffuse across the membrane through transmembrane protein channels (61). The voltage-gated ClC family of chloride channels is widely distributed in prokaryotic and eukaryotic cells and performs diverse physiological roles, including controlling cellular excitability, acidifying intracellular vesicles, and regulating cell volume (62). Acidifying intracellular vesicles benefits the incorporation of iron into the cell (63). Thus, the four genes mentioned above (K7B00_08905, K7B00_06955, K7B00_05320, and K7B00_04890) might play important roles in iron uptake and transport.

Energy is indispensable to cell uptake and transport of metal ions (40). We found that some genes might be related to energy generation during the synthesis of Fe_3_O_4_ nanoparticles, including K7B00_11115, K7B00_05790, K7B00_01245, K7B00_06220, K7B00_12700, and K7B00_13060. Among them, K7B00_11115 and K7B00_05790 encode proteins containing the globin_sensor domain, which are heme-binding sites. It has been reported that MamP and MamT comprising heme-binding sites were necessary for proper magnetite crystallization in *M. magneticum* AMB-1 (64). Therefore, K7B00_11115- and K7B00_05790-encoded proteins might be directly related to electron transfer events in oxidizing or reducing iron mineral intermediates. K7B00_01245 (*hemH*) encodes ferrochelatase, which inserts Fe(II) into porphyrins to complete heme synthesis (65). K7B00_06220 encodes the small subunit of cytochrome *bd* oxidase, which confers bacteria with the ability to maintain aerobic respiration under hostile conditions (66). The cytochrome *bd*-type quinol oxidase has been purified from *A. ferrooxidans* NASF-1 (67). The ferredoxin-like HdrC encoded by K7B00_12700 contains aniron–sulfur (Fe–S) cluster, which was also found in *A. ferrooxidans* LR (68). The NuoF subunit (K7B00_13060) harboring NADH-binding site can provide intracellular NADH output (69). Therefore, these hub genes were mainly related to the oxidation-reduction process, metal ion transport, ferrous chelation, and membrane structure. Interestingly, Fe_3_O_4_ nanoparticle synthesis is closely related to iron redox, but *cyc1*, *cyc2*, *rus*, and other genes that play an important role in electron transport have not been annotated, which need to be further studied.

**Fig 8:**
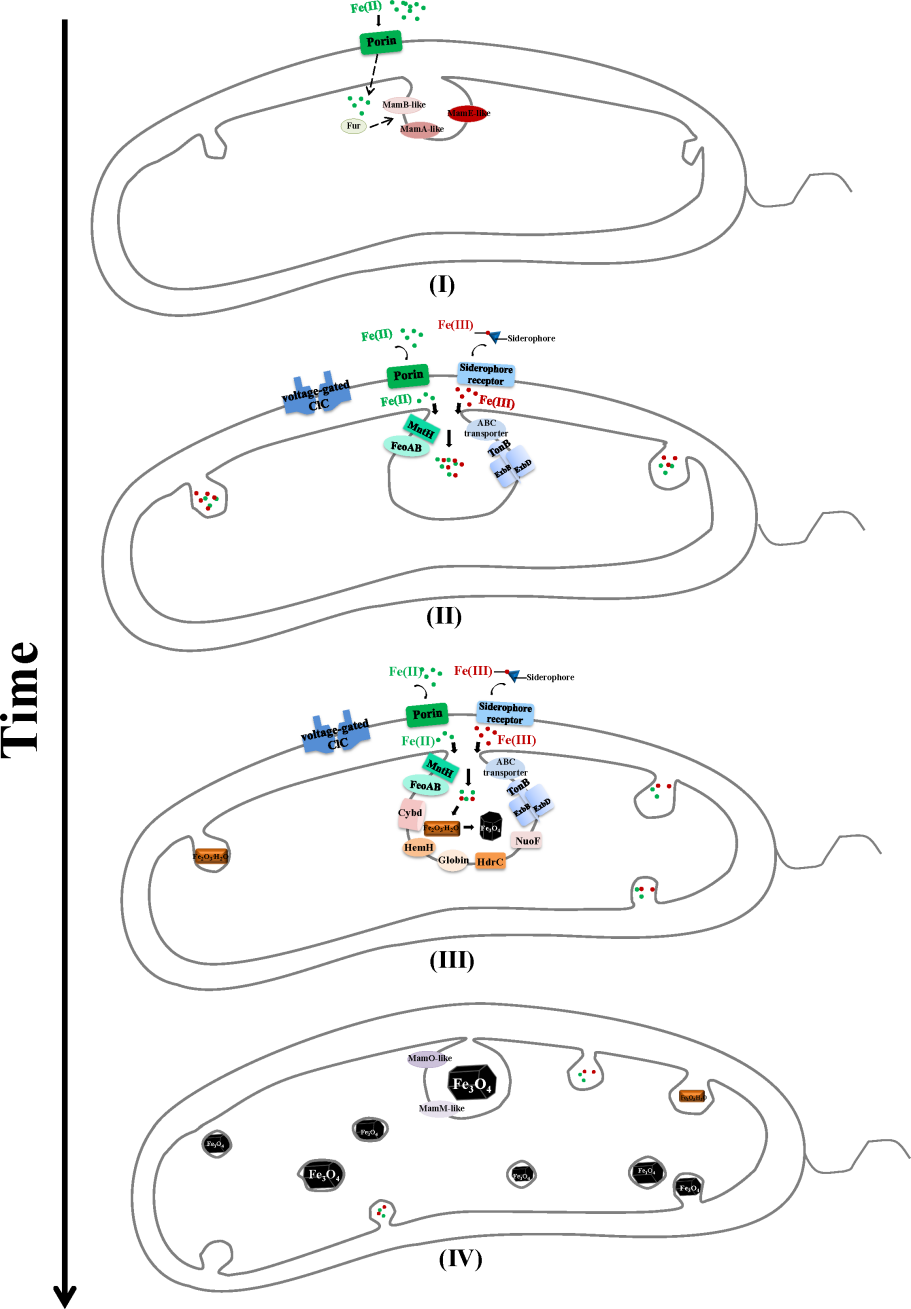
The hypothetical model of Fe_3_O_4_ nanoparticle synthesis in *Acidithiobacillus ferrooxidans* BYM. (I) Membrane formation. Regulated by Fur, the MamA-like, MamB-like, and MamE-like proteins simultaneously initiate the plasma membrane invagination at multiple sites of *A. ferrooxidans* BYM. (II) Iron uptake and transport. Fe(II) and Fe(III) entered the periplasmic space under the action of Porin, MntH, FeoAB, and TonB complex. (III) Iron redox. Under the redox regulation of cytochrome *bd* oxidase, HemH, HdrC, NuoF, and some proteins containing globin_sensor domain, the hydrated ferric oxide (Fe_2_O_3_·nH_2_O) was generated and partially reduced to Fe_3_O_4_. (IV) Crystal mature. The MamM-like and MamO-like proteins might mediate the maturation of Fe_3_O_4_ nanoparticles, which are coated with lipid bilayer membranes.

Interactions between the hub genes determine the function of the enormously complex machinery (70). The magnetosome formation in MTB is a diverse process, which has been studied largely in two model microorganisms, *M. magneticum* AMB-1 and *M. gryphiswaldense* MSR-1. The MAI gene islands mainly control their magnetosome synthesis, and a magnetosome produces at once as each preexisting magnetosome membrane represents a potential site of synthesis (71). However, a magnetosome possibly without membrane encasing produces one at a time from a single magnetosome factory associated with the membrane in *D. magneticus* RS-1 in the absence of MAI (22). Thus, Fe_3_O_4_ nanoparticles coated with lipid bilayers possibly produced from many factories associated with the membrane in *A. ferrooxidans* BYM even though it does not contain MAI in the genome (23, 72). Herein, on the basis of the findings in the present study and the results of previous research (23, 40), we hypothesized that Fe_3_O_4_ nanoparticle synthesis in *A. ferrooxidans* BYM is a complex sequential process mediated by several proteins (Fig. 8).

1. Membrane formation. Fe(II) may enter the periplasmic space through Porin and other iron transporters. The Fur protein could sense the external Fe(II) and may regulate the proteins encoded by *mamA-like*, *mamB-like*, and *mamE-like* genes to simultaneously initiate the plasma membrane invagination at multiple sites of *A. ferrooxidans* BYM (73).
2. Iron uptake and transport. Fe(II) transports from the periplasm to the cytoplasm under the action of MntH (K7B00_06955) and FeoAB. At the same time, the energy provided by the TonB (K7B00_08905) complex promotes the transport of siderophore carrying Fe(III) to the cytoplasm, and the voltage-gated ClC (K7B00_05320, K7B00_04890) may benefit iron uptake.
3. Iron redox. Under the redox regulation of cytochrome *bd* oxidase (K7B00_06220), HemH (K7B00_01245), HdrC (K7B00_12700), NuoF (K7B00_13060), and some proteins containing globin_sensor domain (K7B00_11115, K7B00_05790), the hydrated ferric oxide (Fe_2_O_3_·nH_2_O) was generated and partially reduced to Fe_3_O_4_.
4. Crystal mature. The proteins encoded by *mamM-like* and *mamO-like* genes might mediate the maturation of Fe_3_O_4_ nanoparticles, which are coated with lipid bilayer membranes. This hypothetical molecular model could extend our understanding of the molecular basis of magnetosome synthesis in non-magnetotactic magnetosome-producing bacteria.

### Conclusions

The Fe_3_O_4_ nanoparticle synthesis in *A. ferrooxidans* BYM is a complex, multi-step process involved in several genes. The variation of Fe_3_O_4_ nanoparticle phenomic parameters and transcriptomic data of *A. ferrooxidans* BYM under different treatment conditions were recorded to unravel this complexity. GO and KEGG analyses of the identified DEGs and gene expression profiles highlighted the key biological processes and metabolic pathways involved in Fe_3_O_4_ nanoparticle synthesis. According to gene regulatory networks and co-expression networks, 10 hub genes significantly correlated with phenomic parameters were excavated. A hypothetically sequential molecular model with contributions of hub genes was proposed. However, further investigation is needed to explore the specific function of these key candidate genes experimentally.

## Data Availability

The transcriptome data set of all the samples has been deposited in the NCBI SRA, which is accessible through the accession numbers SRR17069919-SRR17069942. The *A. ferrooxidans* BYM genome alignment can be found in the NCBI BioProject database (BioProject number CP082238).

## SUPPLEMENTAL MATERIAL

Supplementary figures and tables spectrum.01729-23-s0001.pdf Fig. S1 to S16 and Tables S1 to S5.

ASM does not own the copyrights to Supplemental Material that may be linked to, or accessed through, an article. The authors have granted ASM a non-exclusive, world-wide license to publish the Supplemental Material files. Please contact the corresponding author directly for reuse.
