# Supplementary material for: Phenomic and transcriptomic analyses reveal the sequential synthesis of Fe3O4 nanoparticles in Acidithiobacillus ferrooxidans BYM
Source: Microbiol Spectr. 2023 Oct 6;11(6):e01729-23. doi: 10.1128/spectrum.01729-23 (PMC10714799; doi:10.1128/spectrum.01729-23)
Supplement: Supplementary figures and tables — Fig. S1 to S16 and Tables S1 to S5. [file spectrum.01729-23-s0001.pdf]

Figure S1. The principal component analysis of all samples.

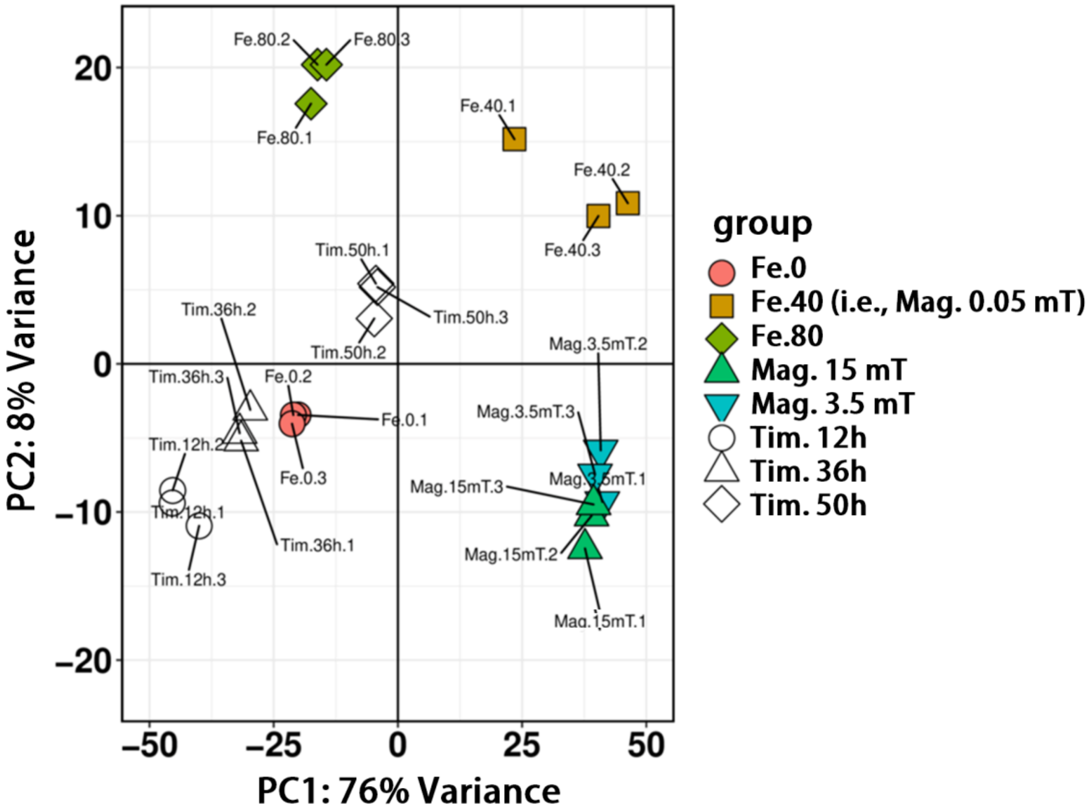

Figure S2. The clustering of DEGs in transcriptome samples cultured in different $FeSO_4 \cdot 7H_2O$ concentrations (A), growth times (B), magnetic field intensities (C).

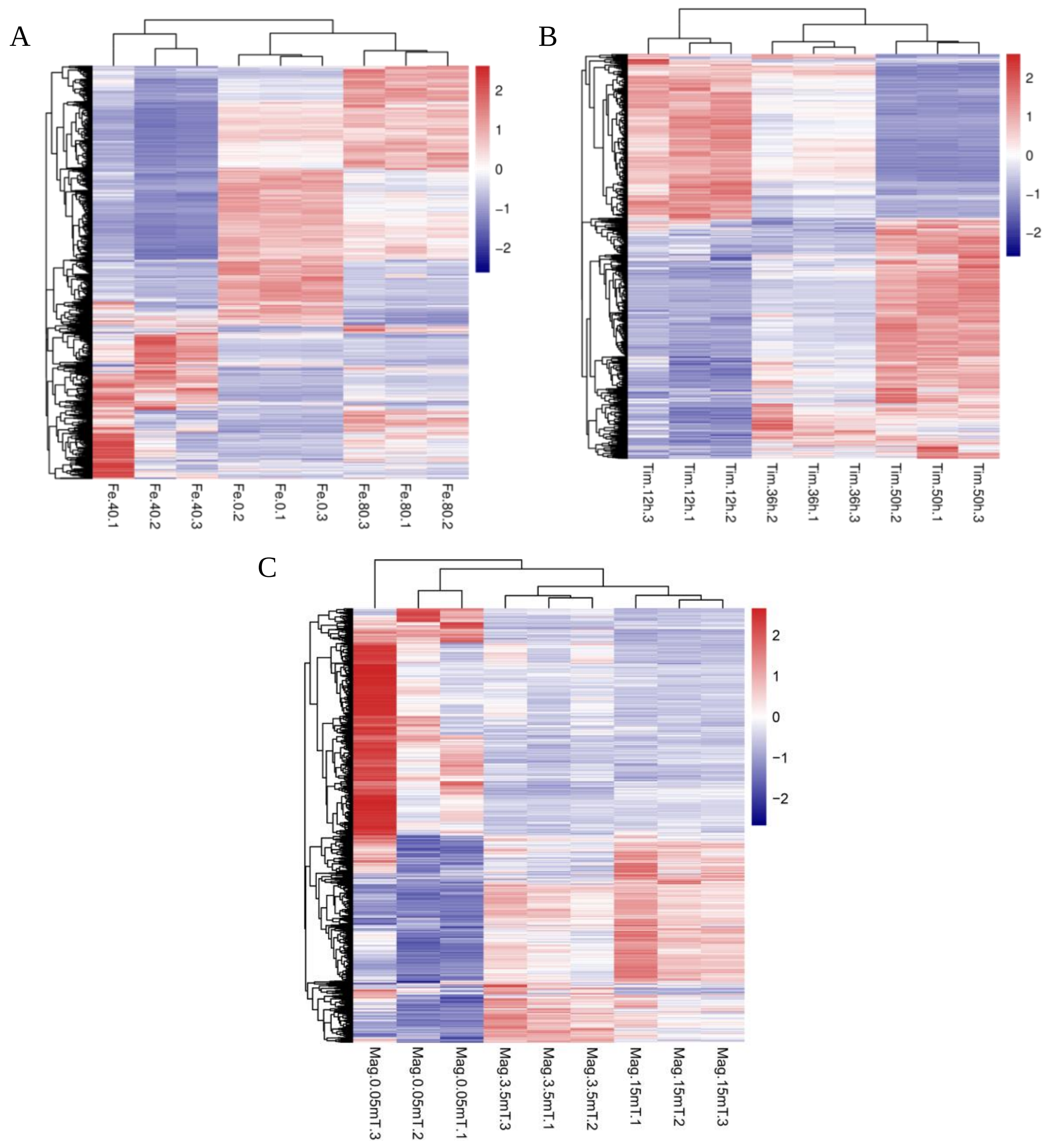

Figure S3. GO enrichment analysis of DEGs in the groups of Fe.0 vs. Fe.40 (A, D, E, F), Fe.80 vs. Fe.40 (B, G, H, I), Fe.0 vs. Fe.80 (C, J, K, L).

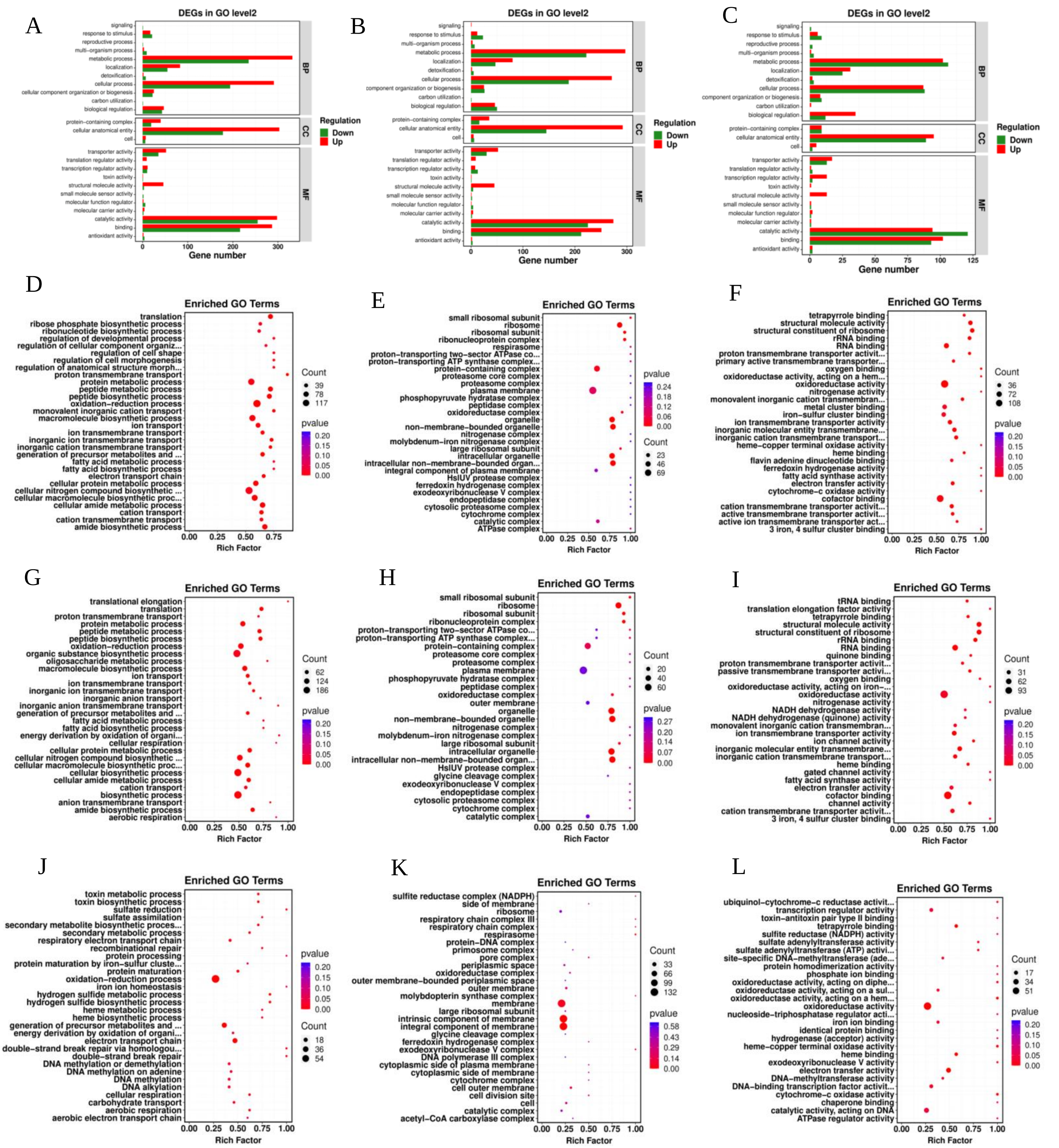

Figure S4. GO enrichment analysis of DEGs in the groups of Tim.12h vs Tim.36h (A, D, E, F), Tim.50h vs Tim.36h (B, G, H, I), Tim.12h vs Tim.50h (C, J, K, L).

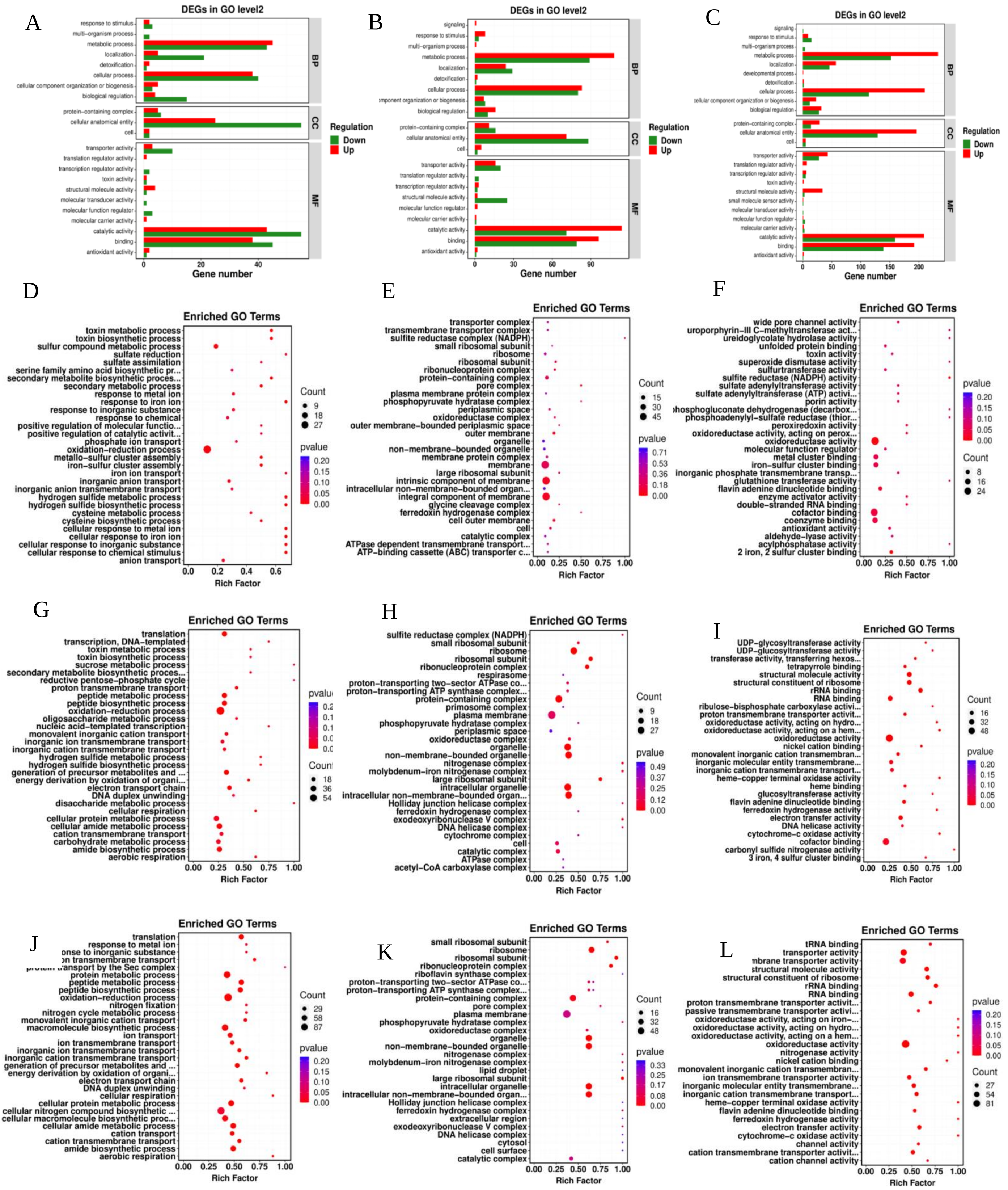

Figure S5. GO enrichment analysis of DEGs in the groups of Mag.3.5mT vs Mag.0.05mT (A, D, E, F), Mag.15mT vs Mag.0.05mT (B, G, H, I), Mag.3.5mT vs Mag.15mT (C, J, K, L).

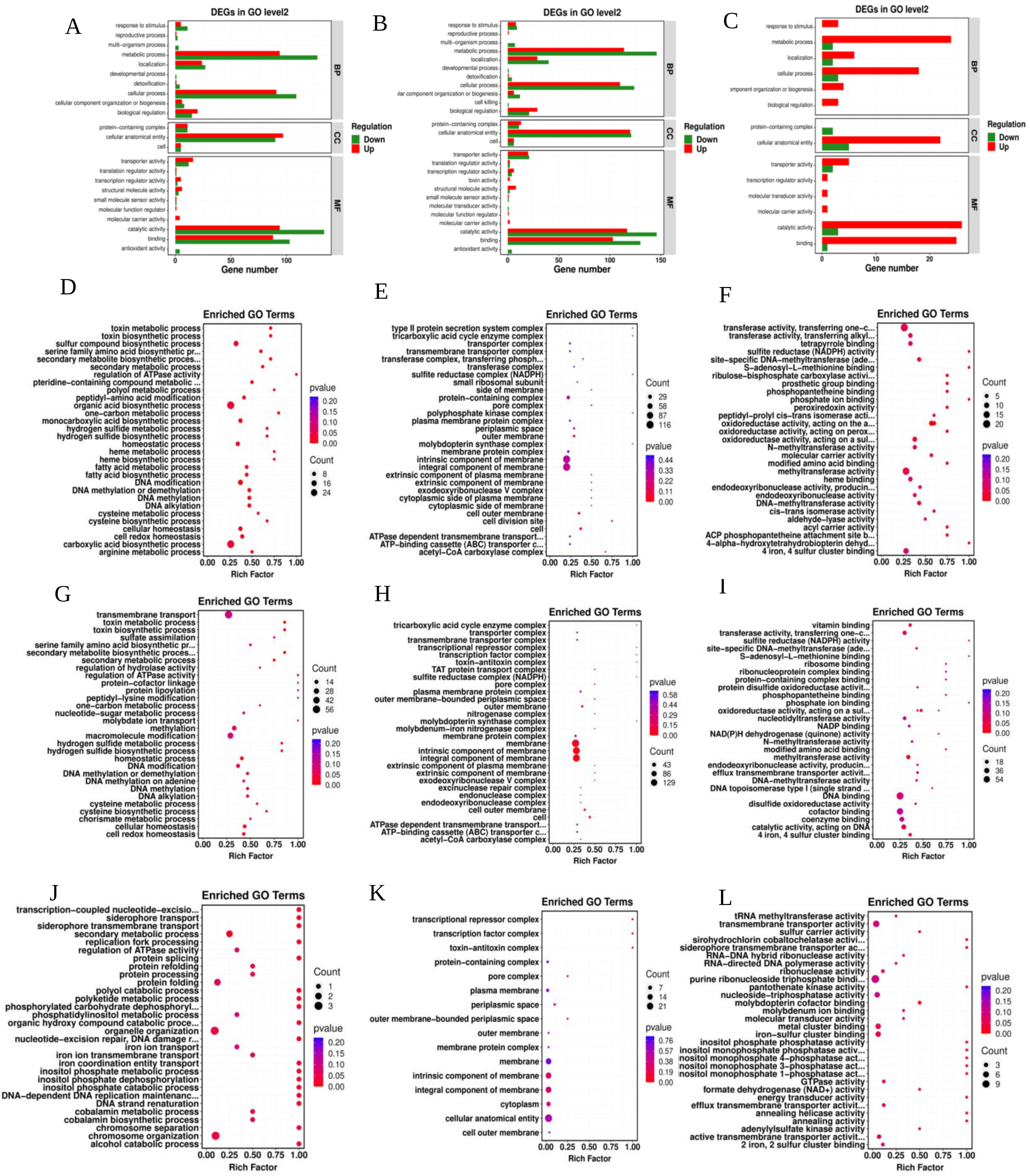

Figure S6. KEGG enrichment analysis of DEGs in the groups of Fe.0 vs. Fe.40 (A, D), Fe.80 vs. Fe.40 (B, E), Fe.0 vs. Fe.80 (C, F).

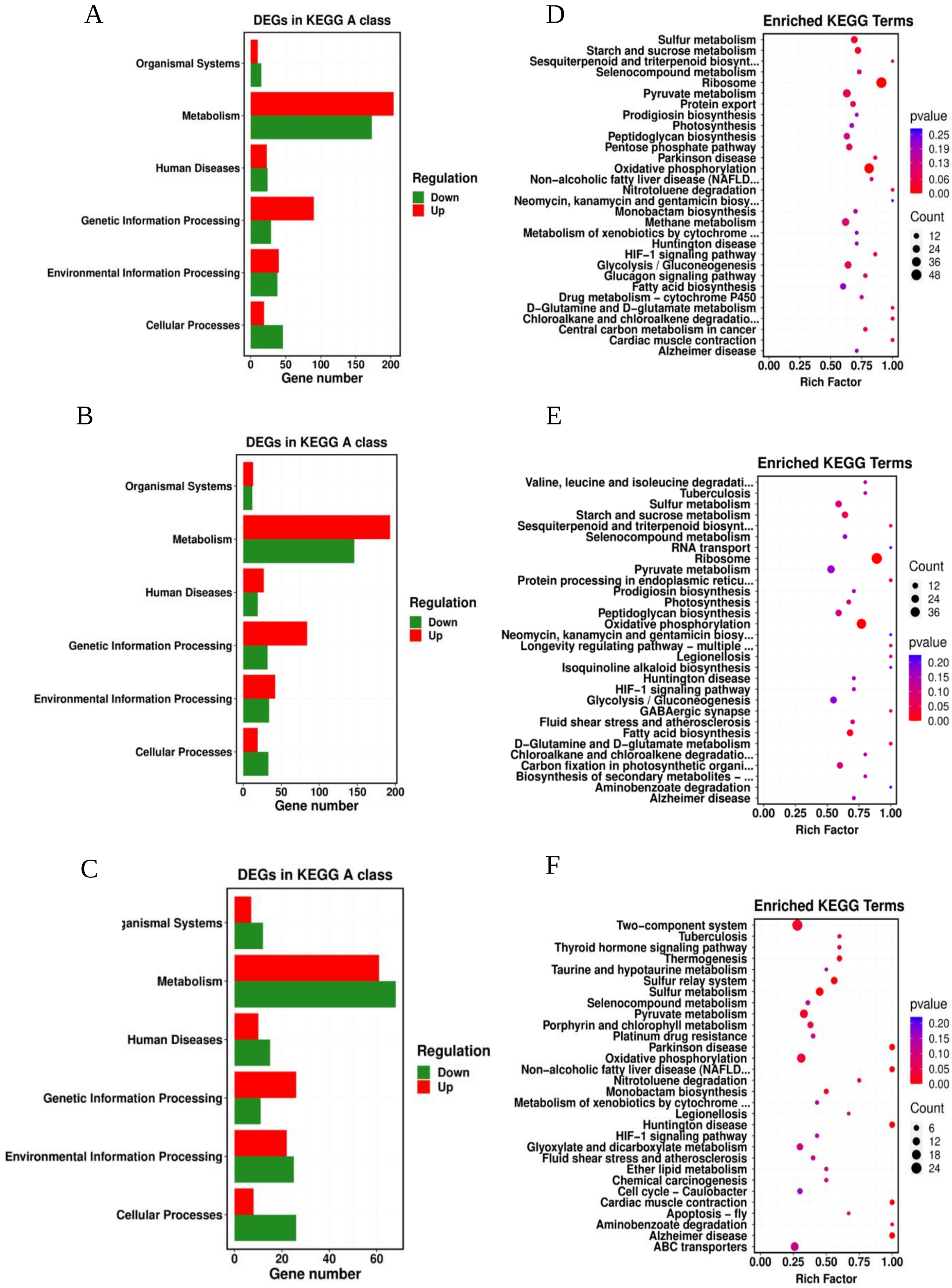

Figure S7. KEGG enrichment analysis of DEGs in the groups of Tim.12h vs Tim.36h (A, D), Tim.50h vs Tim.36h (B, E), Tim.12h vs Tim.50h (C, F).

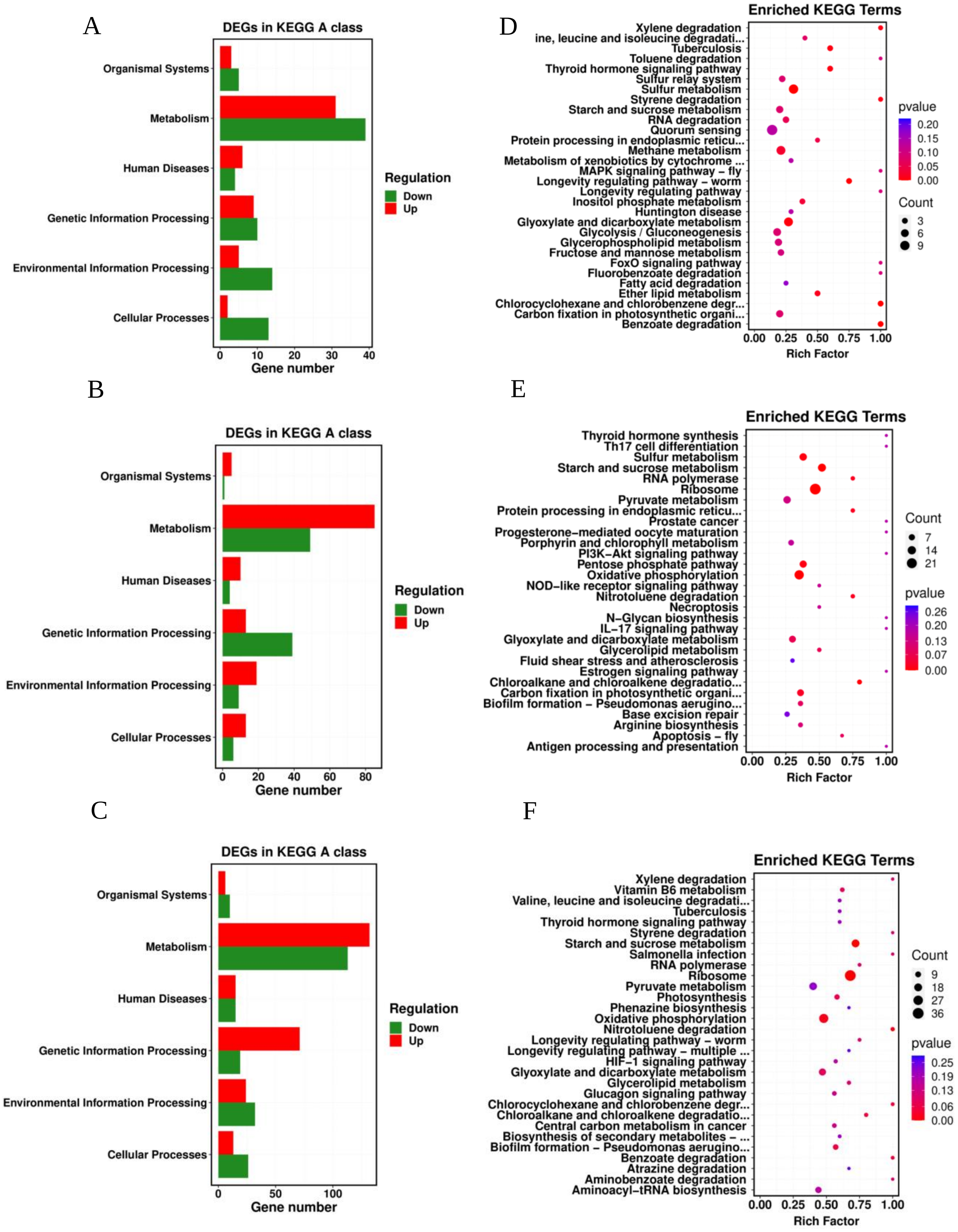

Figure S8. GO enrichment analysis of DEGs in the groups of Mag.3.5mT vs Mag.0.05mT (A, D), Mag.15mT vs Mag.0.05mT (B, E), Mag.3.5mT vs Mag.15mT (C, F).

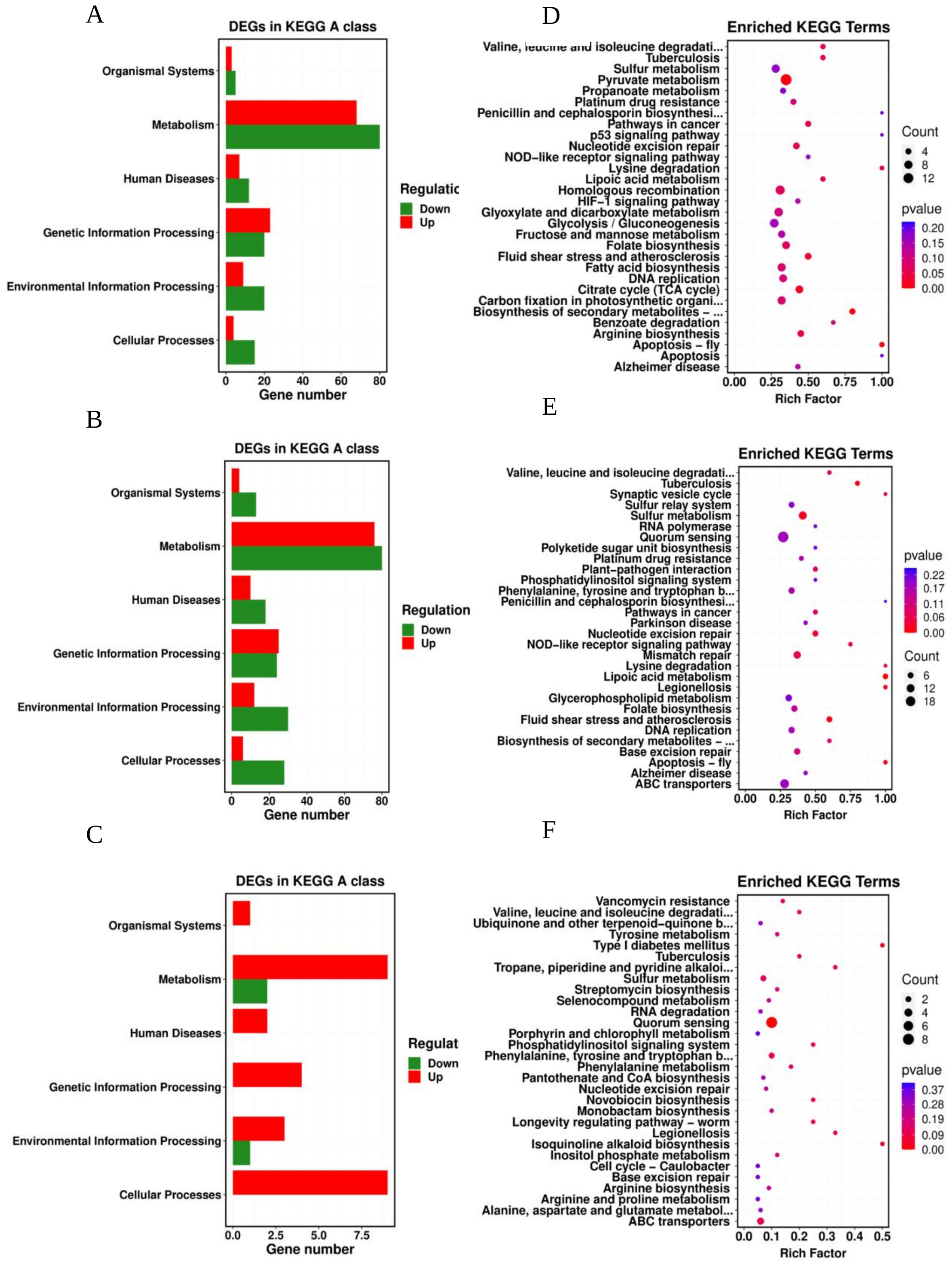

Figure S9. Gene expression trend analyses under different $FeSO_4 \cdot 7H_2O$ concentrations (A), growth times (B), magnetic field intensities (C).

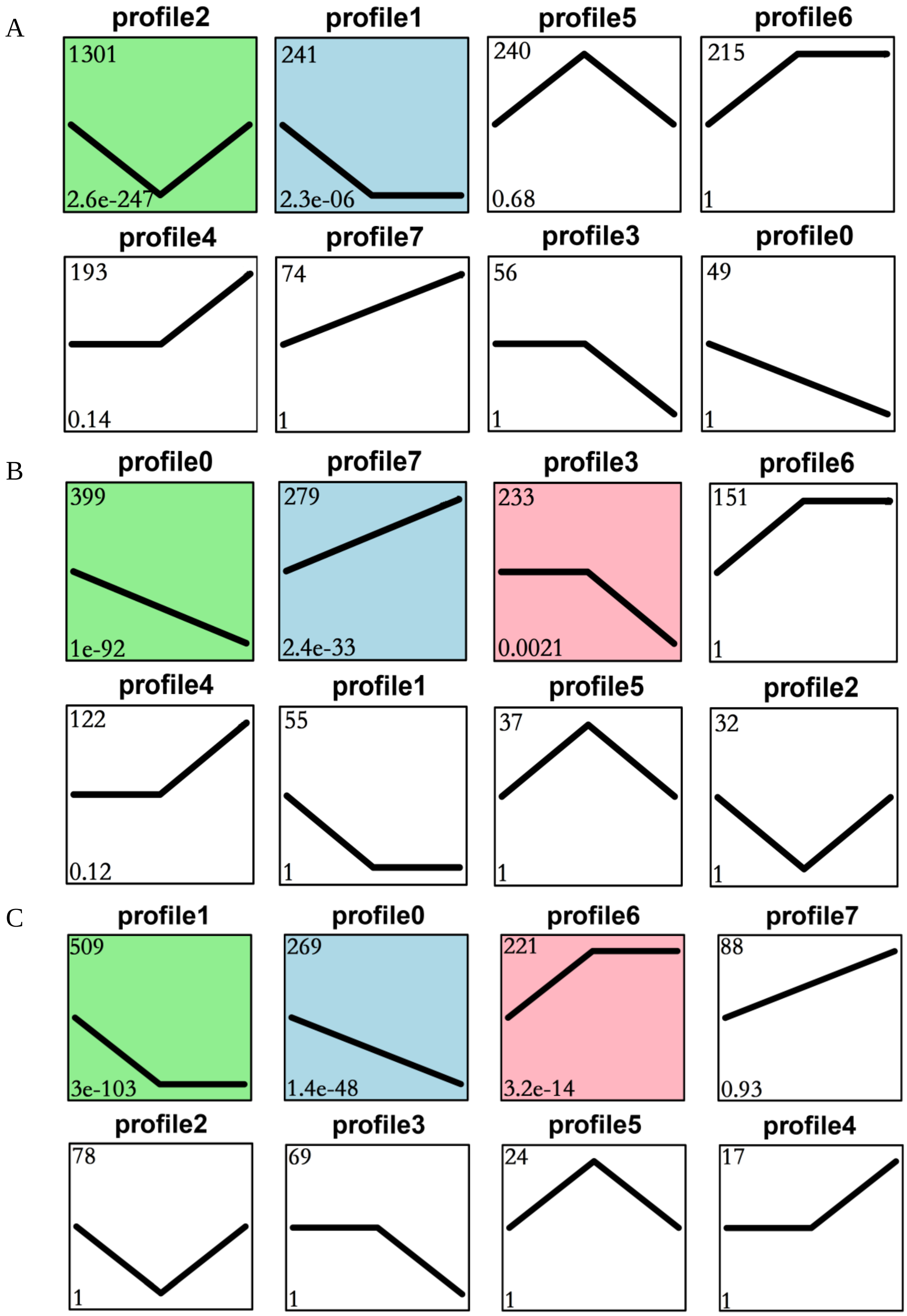

Figure S10. Enrichment analysis of Profile 2 and Profile 1 based on GO (A, B) and KEGG (C, D).

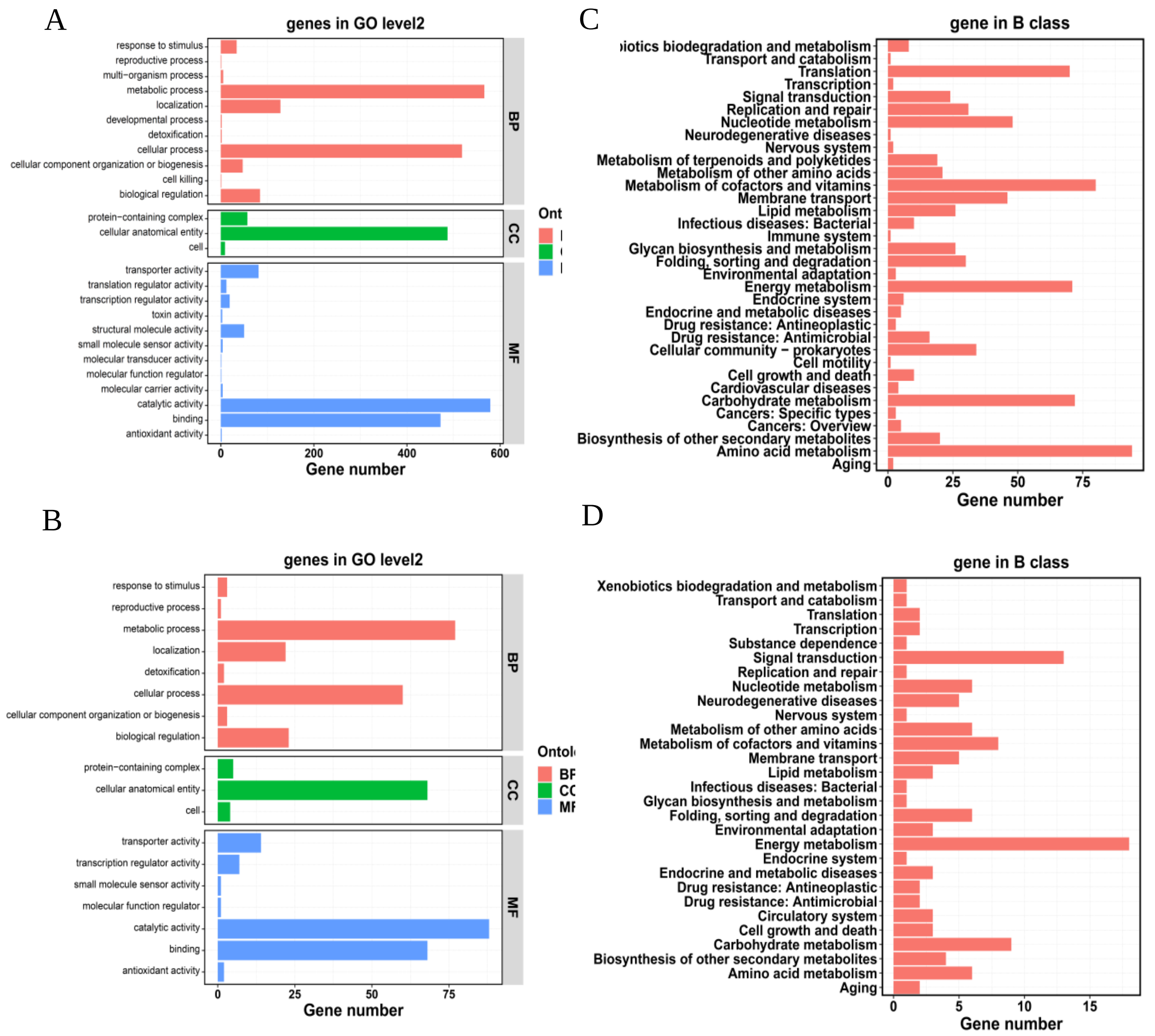

Figure S11. Enrichment analysis of Profile 0, Profile 7 and Profile 3 based on GO (A, B, C) and KEGG (D, E, F).

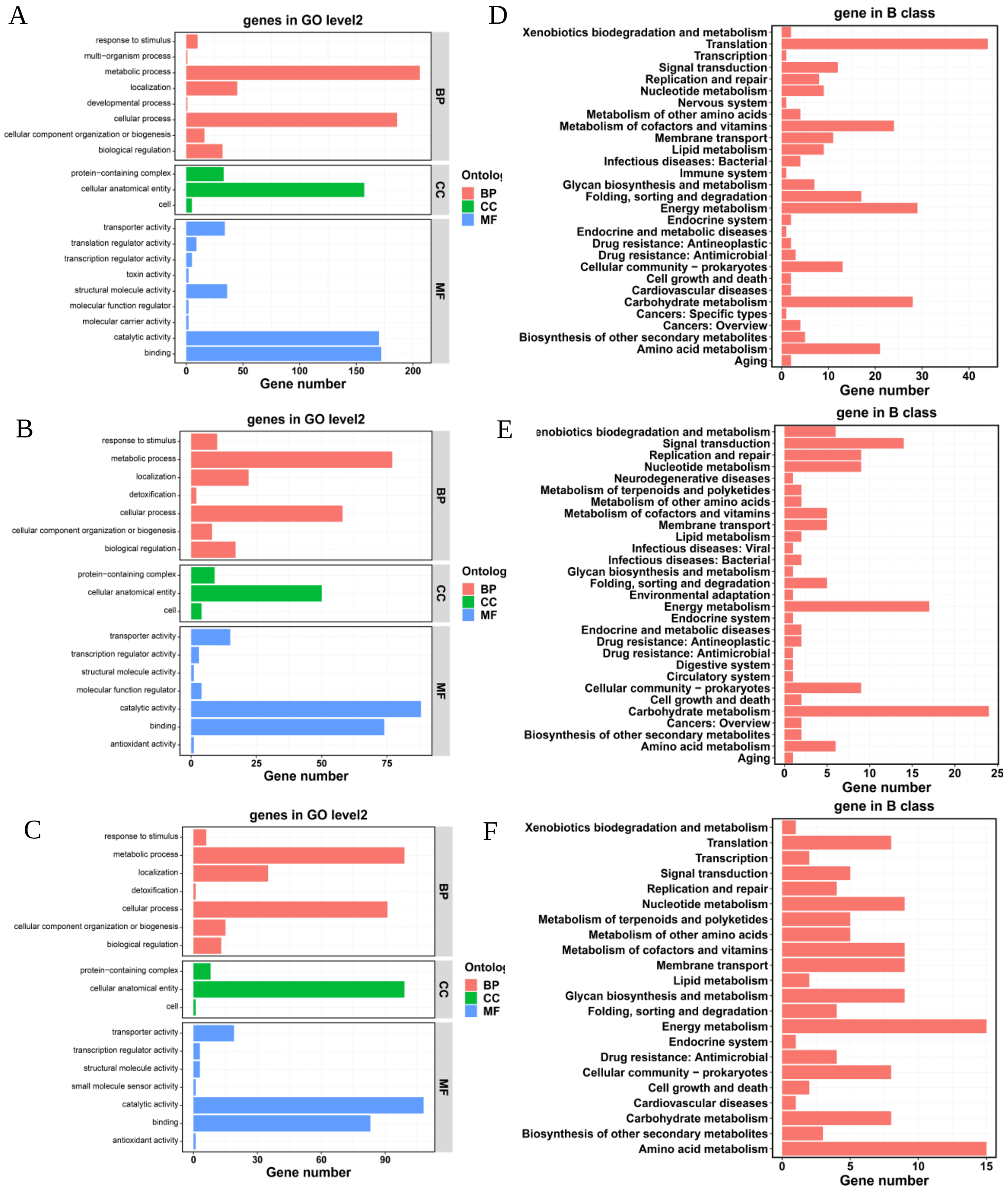

Figure S12. Enrichment analysis of Profile 1, Profile 0 and Profile 6 based on GO (A, B, C) and KEGG (D, E, F).

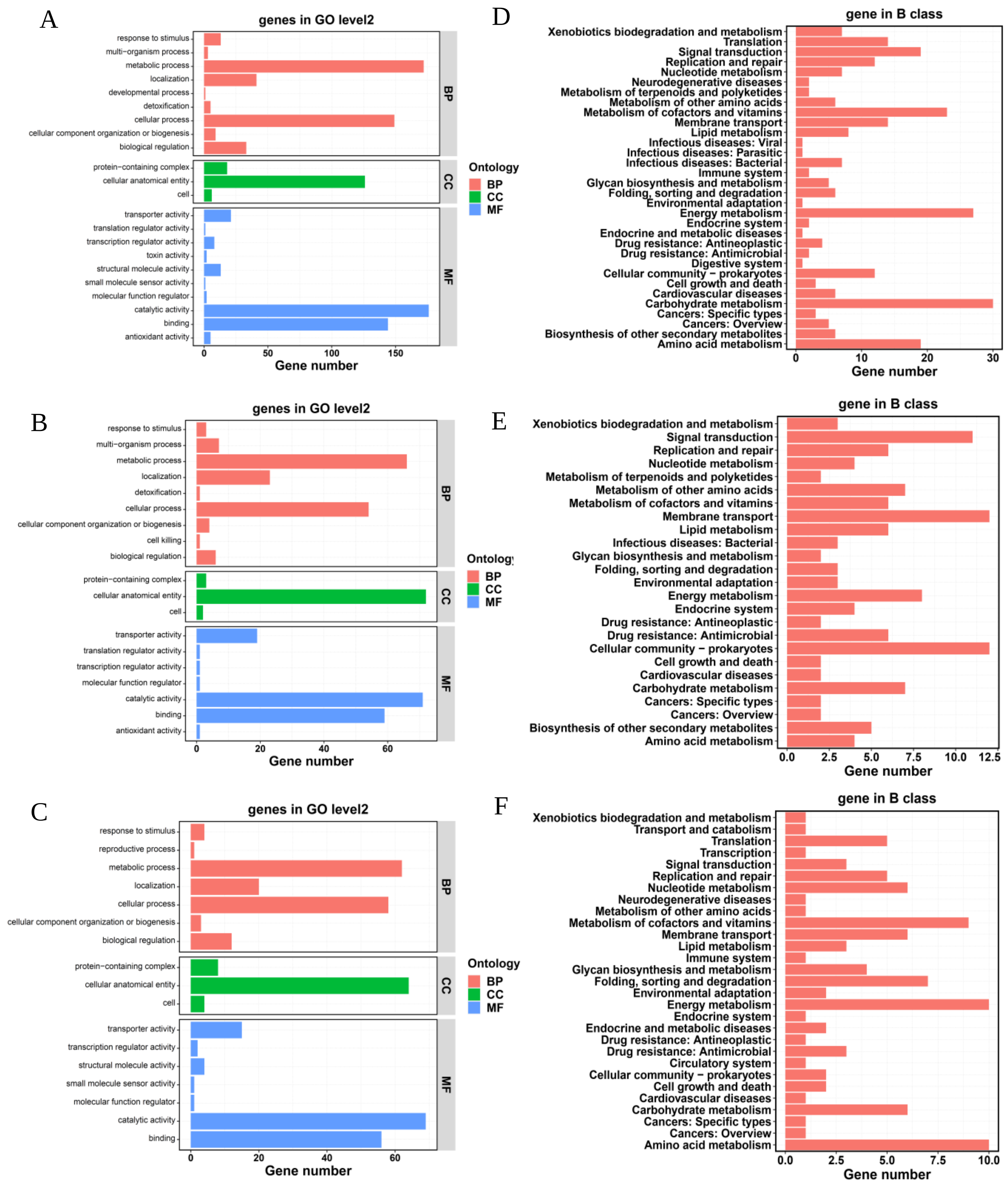

Figure S13. Interaction network of transcription factor.

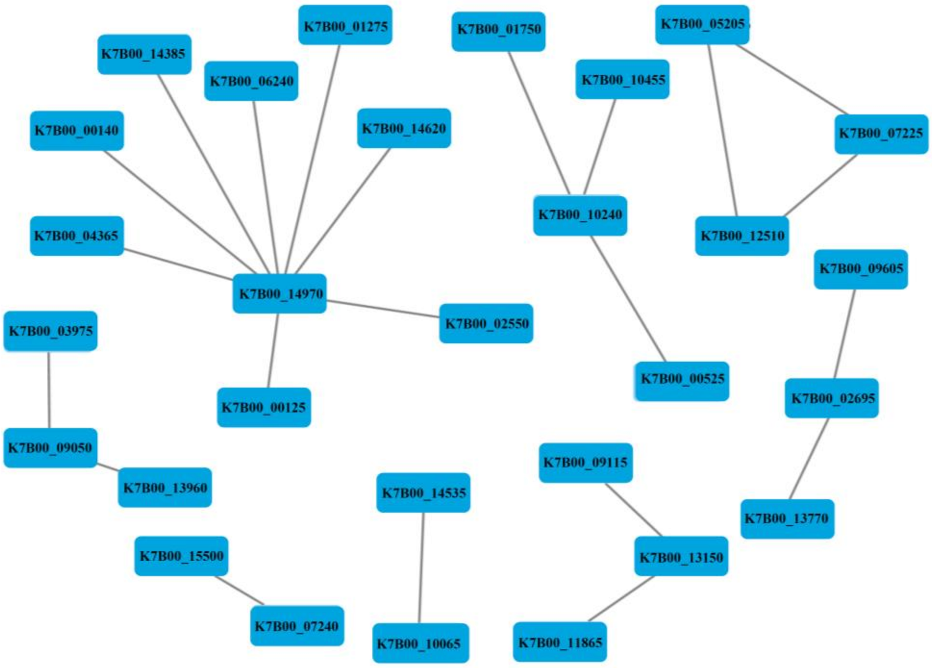

Figure S14. Gene regulatory networks of Fe_3O_4 nanoparticle synthesis in *A. ferrooxidans* BYM.

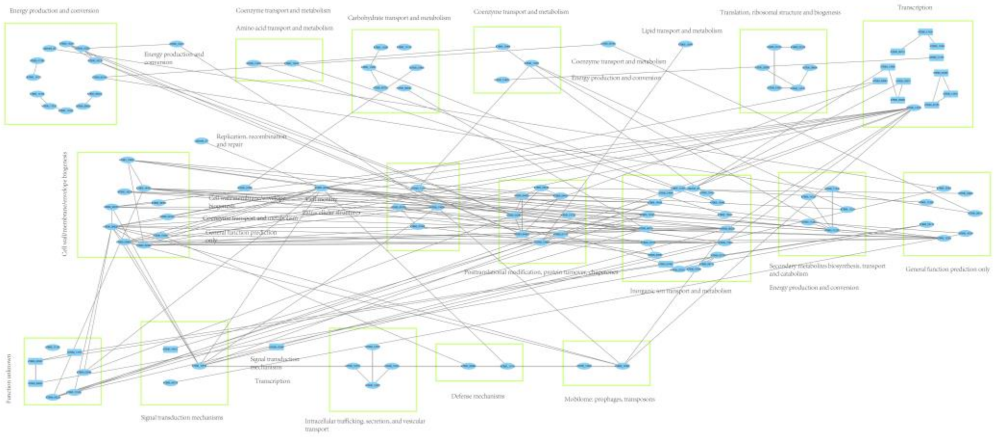

Figure S15. Sample dendrogram and trait heatmap.

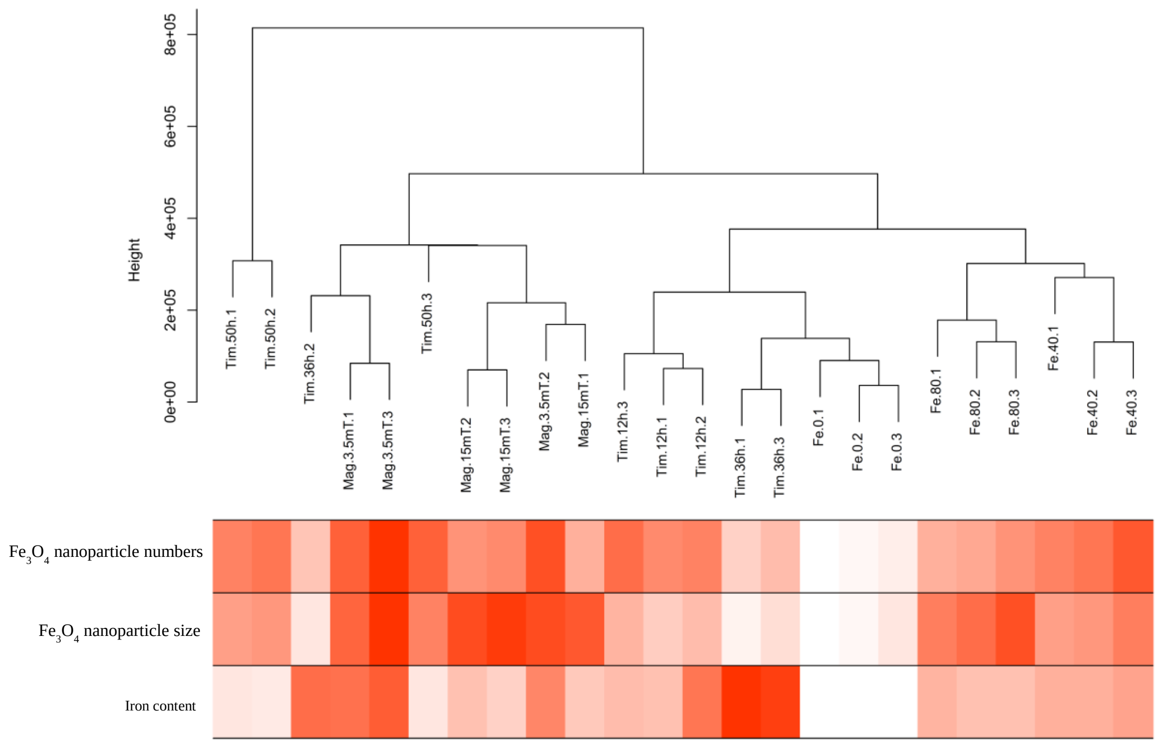

Figure S16. Correlation between expressions of genes related to Fe_3O_4 nanoparticle synthesis and traits.

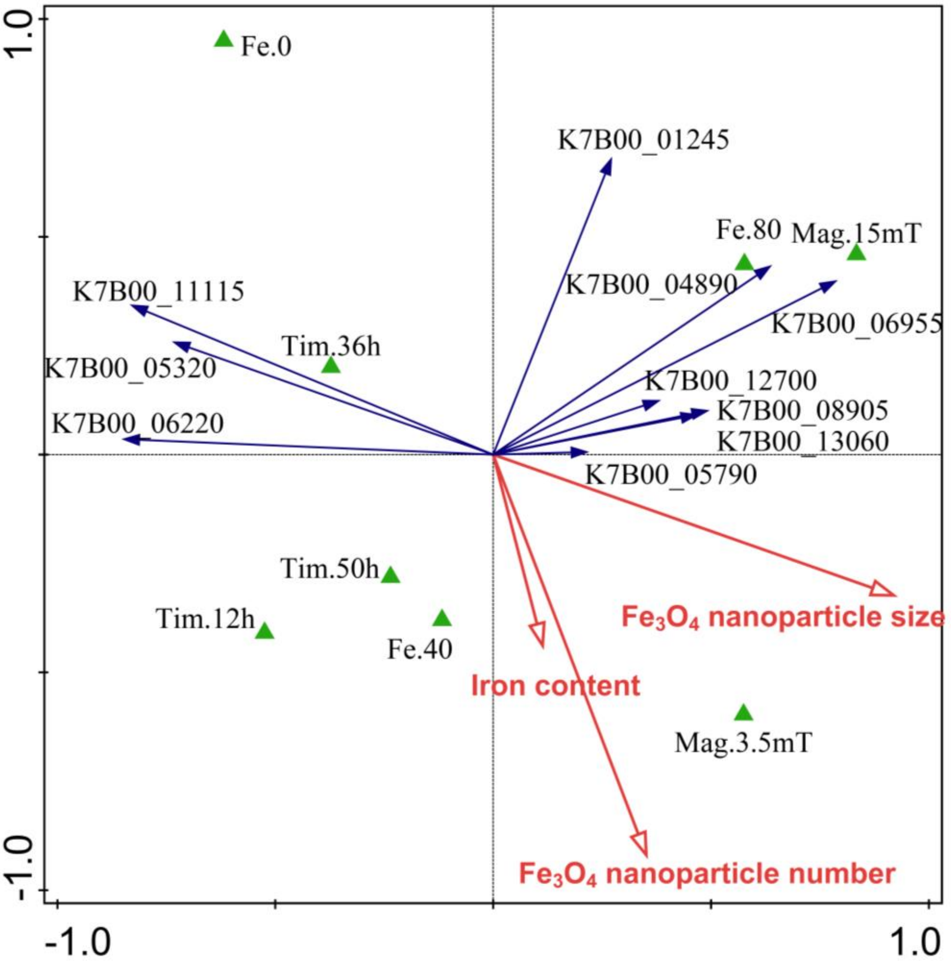

Table S1. Detailed information of each treatment group.

Sample	Treatment condition
Fe.0	aerobically culture in 0K medium supplied with 10 g/L sublimed sulfur under 0.05 mT for 48 h
Fe.40 (i.e., Mag.0.05mT)	aerobically culture in 0K medium supplied with 40 g/L $FeSO_4 \cdot 7H_2O$ under 0.05 mT for 48 h
Fe.80	aerobically culture in 0K medium supplied with 80 g/L $FeSO_4 \cdot 7H_2O$ under 0.05 mT for 48 h
Tim.12	aerobically culture in 0K medium supplied with 40 g/L $FeSO_4 \cdot 7H_2O$ under 0.05 mT for 12 h
Tim.36	aerobically culture in 0K medium supplied with 40 g/L $FeSO_4 \cdot 7H_2O$ under 0.05 mT for 36 h
Tim.50	aerobically culture in 0K medium supplied with 40 g/L $FeSO_4 \cdot 7H_2O$ under 0.05 mT for 50 h
Mag.3.5mT	aerobically culture in 0K medium supplied with 40 g/L $FeSO_4 \cdot 7H_2O$ under 3.5 mT for 48h
Mag.15mT	aerobically culture in 0K medium supplied with 40 g/L $FeSO_4 \cdot 7H_2O$ under 15 mT for 48h

Table S2. Primers used in RT-qPCR.

Gene	Primer	Primer sequences (5'-3')	Tm (^oC)	The length of the product (bp)
16S rRNA	Forward	TGCATGAAGTCGGAATCGCT	61.4	195
	Reverse	GTTCCCCTACGGCTACCTTG	58.9	
K7B00_12700	Forward	CGTCCCGAAGGTGTGATGAA	61.3	161
	Reverse	GCACGTTTGAAGAACCCCAC	60.2	
K7B00_06955	Forward	ATTGCCTCCCATCCACACTG	60.2	120
	Reverse	AATAGTCGTGCCCAGGTTCG	60.0	
K7B00_05790	Forward	CCCATTTTCCTGAAGTGCGG	62.7	149
	Reverse	GATGTGCCCGATACCGAGTT	59.7	
K7B00_01245	Forward	CGCCCCTGATGGTTTACAGT	59.8	228
	Reverse	TGGCAACCGCATCAAAAGTG	62.6	
K7B00_04890	Forward	ATGCCTACAGTCGGAAACGG	60.0	191
	Reverse	CAGCAAAAGCAGCAAGAGCA	60.2	
K7B00_13060	Forward	TGATTATCGGTGCCTACGCC	60.9	153
	Reverse	AGGAGAAATCGGTGCCAAGG	61.4	
K7B00_08905	Forward	GCAGTCAAACAACCGTCCAC	58.4	160
	Reverse	ACTCTGGGTTTAGGTTGCGG	59.3	
K7B00_11115	Forward	AGGTCATCGACCGTTATCGC	59.9	159
	Reverse	CGTGTTCCGTTTGCTTCTGG	61.4	
K7B00_06220	Forward	CGGTCATGGTGTTGTCCCTT	60.2	153
	Reverse	GCTTTCGCTGCTGTTCTTCA	59.1	
K7B00_05320	Forward	CGCCATGTACTCGGTATGCT	58.6	156
	Reverse	CAGAGCCAGGATCAGCAACA	59.2	

Table S3. The data quality of transcriptome sequencing.

Samples	Clean paired reads	Clean bases (G)	Q20 (%)	Q30 (%)	Total mapped (%)
Fe.0.1 (i.e., Mag.0.05mT.1)	7935144	2.20	98.33	94.37	4638165 (64.87)
Fe.0.2 (i.e., Mag.0.05mT.2)	6244434	1.72	98.24	94.12	3449004 (61.05)
Fe.0.3 (i.e., Mag.0.05mT.3)	6855232	1.90	98.47	94.60	4349146 (68.77)
Fe.40.1	3989382	1.15	98.29	94.84	2245944 (70.19)
Fe.40.2	4158484	1.22	98.72	95.76	3125931 (85.10)
Fe.40.3	4153703	1.22	98.63	95.52	2905122 (79.83)
Fe.80.1	7669460	2.17	98.48	95.08	4703164 (77.23)
Fe.80.2	4975881	1.44	98.30	94.83	3014795 (70.47)
Fe.80.3	4256268	1.22	97.97	94.24	2509317 (71.92)
Tim.12h.1	6700188	1.90	97.73	93.18	4222322 (71.81)
Tim.12h.2	6439610	1.81	97.68	93.04	3726642 (69.57)
Tim.12h.3	6354541	1.81	97.92	93.43	4046650 (70.40)
Tim.36h.1	13262186	3.57	98.35	94.83	10145466 (84.51)
Tim.36h.2	15140062	4.07	98.48	95.07	11859000 (86.35)
Tim.36h.3	10823413	2.95	98.48	95.11	8619480 (87.16)
Tim.50h.1	10357369	2.74	97.78	93.46	5634434 (61.17)
Tim.50h.2	9808937	2.62	97.84	93.64	5292017 (60.50)
Tim.50h.3	10059491	2.69	97.84	93.65	5488500 (61.17)
Mag.3.5mT.1	14943612	3.98	97.99	93.75	5989196 (44.00)
Mag.3.5mT.2	14624231	3.89	98.01	93.84	6057886 (45.65)
Mag.3.5mT.3	12160875	3.20	97.88	93.50	4325454 (39.33)
Mag.15mT.1	8798535	2.38	98.14	94.27	5589363 (74.45)
Mag.15mT.2	8408804	2.28	98.29	94.61	5687818 (78.28)
Mag.15mT.3	11720064	3.21	98.24	94.50	7789854 (77.58)

Note: Clean paired reads: The total number of reads after filtering out joints and low-quality bases; Clean bases (G): The total number of filtered bases, which is the number of clean reads multiplied by the length; Q20 (%)、Q30 (%): The percentage of bases with an accuracy of more than 99% or 99.9%; Total mapped (%): The comparison rate of reads and reference genome.

Table S4. NCBI database alignment of partial unaligned reference genomic sequences in Mag.3.5mT to other strains.

Microorganisms	Ratio (%)
Acidithiobacillus ferridurans	0.02
Acidithiobacillus ferrivorans	0.02
Acidithiobacillus ferrivorans SS3	0.02
Acidithiobacillus ferrooxidans	0.21
Acidithiobacillus ferrooxidans ATCC 23270	0.01
Acidithiobacillus ferrooxidans ATCC 53993	0.01
Acidithiobacillus sp. CJ-2	0.01
Acidithiobacillus sp. GGI-221	0.12
Acidithiobacillus sp. SH	0.01
Acidithiobacillus thiooxidans	0.10
Acidiphilium	0.01
Acidiphilium cryptum JF-5	0.01
Acidiphilium sp. 21-60-14	0.18
Homo sapiens	0.05
Gluconacetobacter diazotrophicus PA1 5	0.02
Talaromyces marneffei ATCC 18224	0.02
Bubalus bubalis	0.01
Canislupus familiaris	0.01
Colobusangolensis palliatus	0.01
Mesorhizobium sp.	0.01
Pantroglodytes	0.01
Rhinopithecus roxellana	0.01
Salmonella enterica	0.01
Thiothrix eikelboomii	0.01

Note: The ratio of the number of sequences of other species compared to the number of sequences of unmatched reference genomes.

Table S5. Transcriptional factors in *A. ferrooxidans* BYM.

Gene	class	P2RP description
K7B00_05175	TR	MerR family contains 1 MerR
K7B00_05205	TR	MerR family contains 1 MerR, 1 MerR-DNA-bind
K7B00_00525	RR	OmpR family contains 1 Response_reg, 1 Trans_reg_C
K7B00_05465	TR	ArsR family contains 1 HTH_5
K7B00_05770	OCS	Crp family contains 1 cNMP_binding, 1 HTH_CRP
K7B00_05810	OCS	LysR family contains 1 HTH_1, 1 LysR_substrate
K7B00_05890	OCS	LysR family contains 1 HTH_1, 1 LysR_substrate
K7B00_00575	OCS	Crp family contains 1 cNMP_binding,1 HTH_CRP
K7B00_06105	TR	SfsA family contains 1 SfsA
K7B00_06240	OCS	Unclassified contains 1 PAS_4, 1 AAA_5,1 HTH_11
K7B00_06655	TR	Xre family contains 1 HTH_XRE
K7B00_07070	SF	Ecf family contains 1 Sigma70_r2, 1 Sigma70_r4
K7B00_07225	TR	MerR family contains 1 MerR
K7B00_07240	RR	OmpR family contains 1 Response_reg, 1 Trans_reg_C
K7B00_07400	TR	Fur family contains 1 FUR
K7B00_07930	TR	AbrB family contains 1 SpoVT_AbrB
K7B00_08180	SF	RpoE family contains 1 Sigma70_r1_2, 1 Sigma70_r2, 1 Sigma70_r3, 1 Sigma70_r4
K7B00_08320	OCS	LexA family contains 1 LexA_DNA_bind,1 Peptidase_S24
K7B00_08435	TR	Unclassified contains 1 HTH_12
K7B00_08480	TR	Unclassified contains 1 Bac_DnaA
K7B00_08480	TR	Unclassified contains 1 Bac_DnaA
K7B00_00845	TR	Xre family contains 1 HTH_3
K7B00_08675	TR	Xre family contains 1 HTH_XRE
K7B00_08785	TR	Fur family contains 1 FUR
K7B00_08950	TR	AsnC family contains 1 HTH_DeoR, 1 AsnC_trans_reg
K7B00_08965	OCS	LysR family contains 1 HTH_1, 1 LysR_substrate
K7B00_08975	OCS	LuxR family contains 1 Autoind_bind,1 HTH_LUXR
K7B00_09040	TR	TetR family contains 1 TetR_N
K7B00_09050	TR	MarR family contains 1 MarR
K7B00_09115	TR	ArsR family contains 1 HTH_5
K7B00_09140	RR	OmpR family contains 1 Response_reg, 1 Trans_reg_C
K7B00_09605	TR	AbrB family contains 1 SpoVT_AbrB
K7B00_09700	TR	Xre family contains 1 HTH_3
K7B00_09745	OCS	LysR family contains 1 HTH_1, 1 LysR_substrate
K7B00_09860	TR	MarR family contains 1 MarR
K7B00_00975	TR	Xre family contains 1 HTH_XRE
K7B00_09965	TR	TetR family contains 1 TetR_N
K7B00_10065	RR	OmpR family contains 1 Response_reg, 1 Trans_reg_C
K7B00_10240	TR	Unclassified contains 1 Trans_reg_C
K7B00_10320	OCS	GntR family contains 1 GntR, 1 UTRA

K7B00_10405	RR	OmpR family contains 1 Response_reg, 1 Trans_reg_C
K7B00_10455	RR	OmpR family contains 1 Response_reg, 1 Trans_reg_C
K7B00_10525	SF	RpoE family contains 1 Sigma70_r1_1, 1 Sigma70_r1_2, 1 Sigma70_ner, 1 Sigma70_r2, 1 Sigma70_r3, 1 Sigma70_r4
K7B00_10550	SF	Ecf family contains 1 Sigma70_r2, 1 Sigma70_r4
K7B00_10565	OCS	LysR family contains 1 HTH_1, 1 LysR_substrate
K7B00_11025	TR	Xre family contains 1 HTH_XRE
K7B00_11055	TR	MarR family contains 1 MarR
K7B00_11195	OCS	LysR family contains 1 HTH_1, 1 LysR_substrate
K7B00_11310	TR	IclR family contains 1 HTH_IclR, 1 IclR
K7B00_11355	OCS	Crp family contains 1 cNMP_binding, 1 HTH_CRP
K7B00_11475	TR	Xre family contains 1 HTH_XRE
K7B00_11865	TR	ArsR family contains 1 HTH_5
K7B00_12095	SF	RpoE family contains 1 Sigma70_r1_2, 1 Sigma70_r2, 1 Sigma70_r3, 1 Sigma70_r4
K7B00_01210	OCS	LysR family contains 1 HTH_1, 1 LysR_substrate
K7B00_12215	TR	Crp family contains 1 HTH_CRP
K7B00_12260	RR	OmpR family contains 1 Response_reg, 1 Trans_reg_C
K7B00_00125	RR	NtrC family contains 1 Response_reg, 1 AAA_5, 1 HTH_8
K7B00_12510	TR	MerR family contains 1 MerR
K7B00_12575	TR	FeoC family contains 1 FeoC
K7B00_12605	OCS	LysR family contains 1 HTH_1, 1 LysR_substrate
K7B00_01275	RR	NtrC family contains 1 Response_reg, 1 AAA_5, 1 HTH_8
K7B00_12920	TR	HxlR family contains 1 HxlR
K7B00_13150	TR	ArsR family contains 1 HTH_5
K7B00_13270	TR	HrcA family contains 1 HTH_11, 1 HrcA
K7B00_01340	TR	HxlR family contains 1 HxlR
K7B00_13660	SF	Ecf family contains 1 Sigma70_r1_2, 1 Sigma70_r2, 1 Sigma70_r4
K7B00_13770	TR	AbrB family contains 1 SpoVT_AbrB
K7B00_13900	TR	Crp family contains 1 HTH_CRP
K7B00_13915	OCS	AraC family contains 1 Ada_Zn_binding, 1 HTH_AraC, 1 AlkA_N, 1 HhH-GPD
K7B00_00140	RR	NtrC family contains 1 Response_reg, 1 AAA_5, 1 HTH_8
K7B00_14185	TR	ArsR family contains 1 HTH_5
K7B00_14315	TR	AbrB family contains 1 SpoVT_AbrB
K7B00_14330	TR	AbrB family contains 1 SpoVT_AbrB
K7B00_14385	RR	NtrC family contains 1 Response_reg, 1 AAA_5, 1 HTH_8
K7B00_14535	TR	Unclassified contains 1 Trans_reg_C
K7B00_14620	RR	NtrC family contains 1 Response_reg, 1 AAA_5, 1 HTH_8
K7B00_14750	SF	Unclassified contains 1 Sigma54_DBD
K7B00_14970	SF	RpoN family contains 1 Sigma54_AID, 1 Sigma54_CBD,1 Sigma54_DBD
K7B00_15500	RR	PrrA family contains 1 Response_reg, 1 HTH_8

K7B00_15515	TR	Rrf2 family contains 1 Rrf2
K7B00_16005	TR	Rrf2 family contains 1 Rrf2
K7B00_01620	TR	TetR family contains 1 TetR_N
K7B00_16165	RR	OmpR family contains 1 Response_reg, 1 Trans_reg_C
K7B00_01695	OCS	Crp family contains 1 cNMP_binding, 1 HTH_CRP
K7B00_01750	TR	Fur family contains 1 FUR
K7B00_01820	TR	NrdR family contains 1 nrdR
K7B00_00185	OCS	LysR family contains 1 HTH_1, 1 LysR_substrate
K7B00_02550	RR	NtrC family contains 1 Response_reg, 1 AAA_5, 1 HTH_8
K7B00_02660	SF	RpoE family contains 1 Sigma70_r2, 1
K7B00_02695	TR	AbrB family contains 1 SpoVT_AbrB
K7B00_00375	TR	MarR family contains 1 MarR
K7B00_03975	TR	Xre family contains 1 HTH_XRE
K7B00_04220	TR	Rrf2 family contains 1 Rrf2
K7B00_04360	OCS	LysR family contains 1 HTH_1, 1 LysR_substrate
K7B00_04365	RR	NtrC family contains 1 Response_reg, 1 AAA, 1 HTH_8
K7B00_00445	TR	Xre family contains 1 HTH_XRE
K7B00_04900	OCS	LysR family contains 1 HTH_1, 1 LysR_substrate
K7B00_04940	TR	PadR family contains 1 PadR
plasmid_27	OCS	Crp family contains 1 cNMP_binding, 1 HTH_CRP
plasmid_6	TR	LysR family contains 1 HTH_1
